# Identification of hub genes in hepatocellular carcinoma using integrated bioinformatic analysis

**DOI:** 10.18632/aging.102969

**Published:** 2020-03-26

**Authors:** Shengni Hua, Zhonghua Ji, Yingyao Quan, Meixiao Zhan, Hao Wang, Wei Li, Yong Li, Xu He, Ligong Lu

**Affiliations:** 1 Zhuhai Interventional Medical Center, Zhuhai Precision Medical Center, Zhuhai People’s Hospital, Zhuhai Hospital Affiliated with Jinan University, Zhuhai 519000, China; 2 Department of Anesthesiology, Zhuhai People’s Hospital, Zhuhai Hospital Affiliated with Jinan University, Zhuhai 519000, China

**Keywords:** hepatocellular carcinoma, hub genes, bioinformatic analysis, prognostic value, diagnostic value

## Abstract

The molecular mechanisms underlying hepatocellular carcinoma (HCC) progression remain largely undefined. Here, we identified 176 commonly upregulated genes in HCC tissues based on three Gene Expression Omnibus datasets and The Cancer Genome Atlas (TCGA) cohort. We integrated survival and methylation analyses to further obtain 12 upregulated genes for validation. These genes were overexpressed in HCC tissues at the transcription and protein levels, and increased mRNA levels were related to higher tumor grades and cancer stages. The expression of all markers was negatively associated with overall and disease-free survival in HCC patients. Most of these hub genes can promote HCC proliferation and/or metastasis. These 12 hub genes were also overexpressed and had strong prognostic value in many other cancer types. Methylation and gene copy number analyses indicated that the upregulation of these hub genes was probably due to hypomethylation or increased gene copy numbers. Further, the methylation levels of three genes, *KPNA2*, *MCM3*, and *LRRC1*, were associated with HCC clinical features. Moreover, the levels of most hub genes were related to immune cell infiltration in HCC microenvironments. Finally, we identified three upregulated genes (*KPNA2*, *TARBP1*, and *RNASEH2A*) that could comprehensively and accurately provide diagnostic and prognostic value for HCC patients.

## INTRODUCTION

Liver cancer is one of the greatest threats to human health worldwide, with hepatocellular carcinoma (HCC) accounting for approximately 80% of these cases [1]. Due to its complex pathogeny, heterogeneity of cancer cells, and low rates of diagnosis at early stages, the treatment of this disease is still not optimal and patient prognosis is poor [2]. Despite extensive high-throughput sequencing technologies and microarray analyses to clarify molecular targets associated with the progression of HCC, small sample sizes in individual studies and the diversity of technology platforms utilized have led to substantial discrepancies in the research, making statistical analyses difficult. Recently, integrated bioinformatics methods and data reanalysis have been used to overcome this problem [3–5].

The role of epigenetics in cancer progression has attracted increasing attention. Abnormal DNA methylation can modulate the expression of cancer-related genes at the transcriptional level and predict prognosis for patients [6]. As an important epigenetic regulatory mechanism, DNA methylation is involved in the modulation of cancer cell proliferation, apoptosis, and invasion [7–9]. Although some research has shown that the abnormal methylation of certain genes affects HCC cell senescence, tumor growth, metastasis, hepatic carcinogenesis, and patient prognosis [10–12], a comprehensive integrative analysis of such gene networks has not been performed.

Genomic DNA copy number can affect gene expression and is involved in cancer progression. Alterations to copy number signatures can lead to transcriptome imbalances and the abnormal expression of cancer-related genes, which can be used to predict both overall survival and sensitivity to drug treatment [2, 13]. Therefore, a combination of the information from DNA copy number arrays, DNA methylation arrays, mRNA detection, and protein level detection can sufficiently clarify the molecular mechanisms and predict novel treatment targets for HCC.

In this study, The Cancer Genome Atlas (TCGA) HCC cohort and Gene Expression Omnibus (GEO) datasets were used to identify differentially-expressed genes (DEGs) between HCC and normal tissues. Twelve upregulated hub genes were selected to detect the association between their expression and clinical prognosis. Moreover, their methylation levels, gene copy numbers, and relationships with immune cell infiltration were assessed. Finally, we identified three upregulated genes that could serve as diagnostic and prognostic indicators for HCC patients.

## RESULTS

### Identification of DEGs in HCC

**Figure 1:**
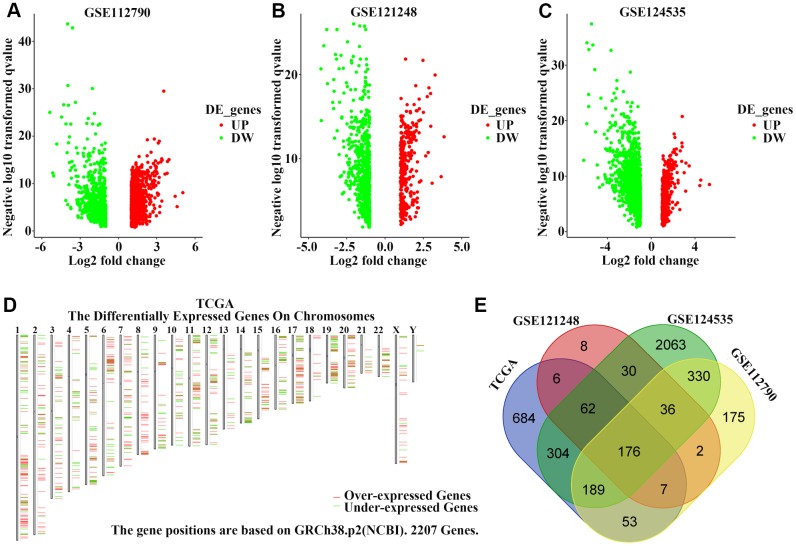
**Identification of upregulated genes in hepatocellular carcinoma (HCC) tissues.** (**A**–**C**) Volcano plot visualizing the differentially-expressed genes between HCC and non-tumor tissues in (**A**) GSE112790, (**B**) GSE121248, and (**C**) GSE124535 datasets. Each symbol represents a gene, and red or green colors indicate upregulated or downregulated genes, respectively. (**D**) The specific chromosomal locations of differentially-expressed genes between HCC and non-tumor tissues in the TCGA cohort. Red indicates overexpressed genes and green indicates downregulated genes. The vertical line represents chromosomes. (**E**) Common upregulated genes among GSE112790, GSE121248, GSE124535, and TCGA datasets.

We first obtained the gene expression information for HCC and non-tumor liver tissues from three GEO datasets and a TCGA cohort and performed data pre-procession using GEO2R and GEPIA2 websites to screen DEGs based on the cut-off criteria of a *p*-value < 0.01 and [log_2_FC (fold-change)] > 1. Figure 1A–1C shows the DEGs from GSE112790, GSE121248, and GSE124535 databases, respectively, based on a volcano plot. We also identified DEGs in the TCGA cohort and visualized their chromosomal locations (Figure 1D). These DEGs were distributed in all chromosomes. A total of 176 upregulated genes (Supplementary Table 1) were found to be common to all four datasets (Figure 1E).

### Functional enrichment analysis, PPI network construction, and module analysis

**Figure 2:**
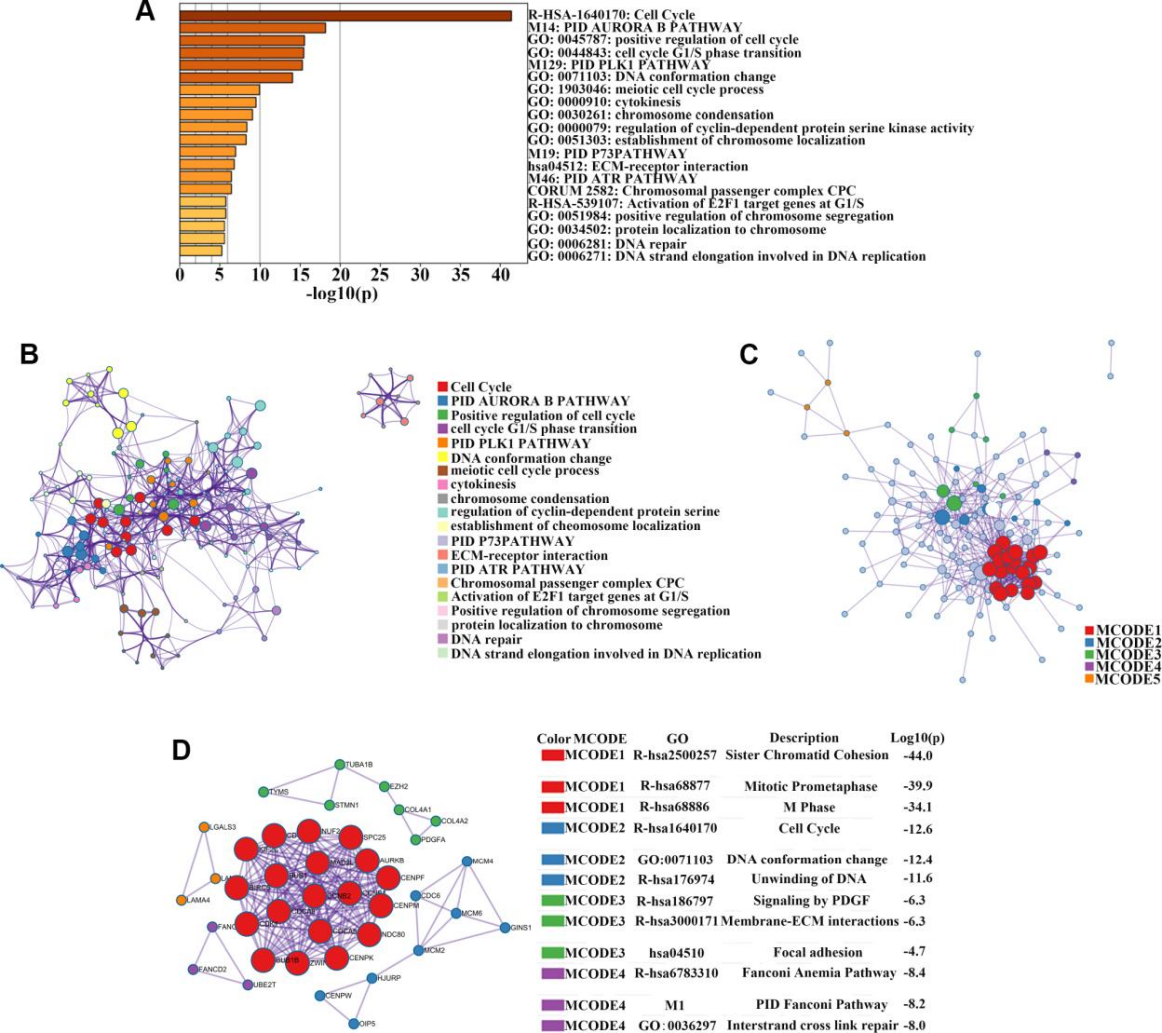
**Enrichment analysis, protein–protein interaction (PPI) network construction, and module analysis.** (**A**) Metascape bar graph to view the top 20 non-redundant enrichment clusters of upregulated genes. The enriched biological processes were ranked by *p*-value. A deeper color indicates a smaller *p*-value. (**B**) Metascape visualization of the networks of the top 20 clusters. Each node represents one enriched term colored by cluster ID; nodes that share the same cluster are typically close to each other. Node size is proportional to the number of input genes falling into that term. Thicker edges indicate higher similarity. (**C**) PPI network construction of upregulated genes. (**D**) Four sub-networks were identified by Cytoscape MCODE plug-in analysis. Ingenuity pathway analysis of genes in each sub-network to obtain the biological pathways.

To investigate the biological significance of these overlapping genes, we uploaded the list of 176 upregulated genes into Metascape software for functional enrichment analysis. Upregulated genes were mainly enriched in biological processes such as cell cycle, chromosome condensation, and DNA replication, as well as molecular functions such as protein serine kinase activity, ECM-receptor interaction, and activation of E2F1 target genes. Most enriched clusters were associated with cancer. Figure 2A–2B shows the top 20 clusters of significantly-enriched terms. Next, Metascape and Cytoscape (v3.1.2) were used to construct the PPI network of the 176 upregulated genes (Figure 2C). We identified four significant modules by performing cluster analysis of the PPI network with the Cytoscape MCODE plug-in based on the degree of importance (Figure 2D). We then performed GO and pathway enrichment analyses of the genes in these modules (Figure 2D). The genes in Module 1 were mainly enriched in sister chromatid cohesion, mitotic prometaphase, and M phase. Genes in Module 2 were mainly correlated with cell cycle, DNA conformation change, and unwinding of DNA. Genes in Module 3 were mainly enriched in signaling by PDGF, membrane-ECM interactions, and focal adhesion. Genes in Module 4 were mainly correlated with the Fanconi anemia pathway, pid Fanconi pathway, and interstrand cross-link repair (Figure 2D).

### Identification of HCC prognosis-related genes with lower methylation levels

**Figure 3:**
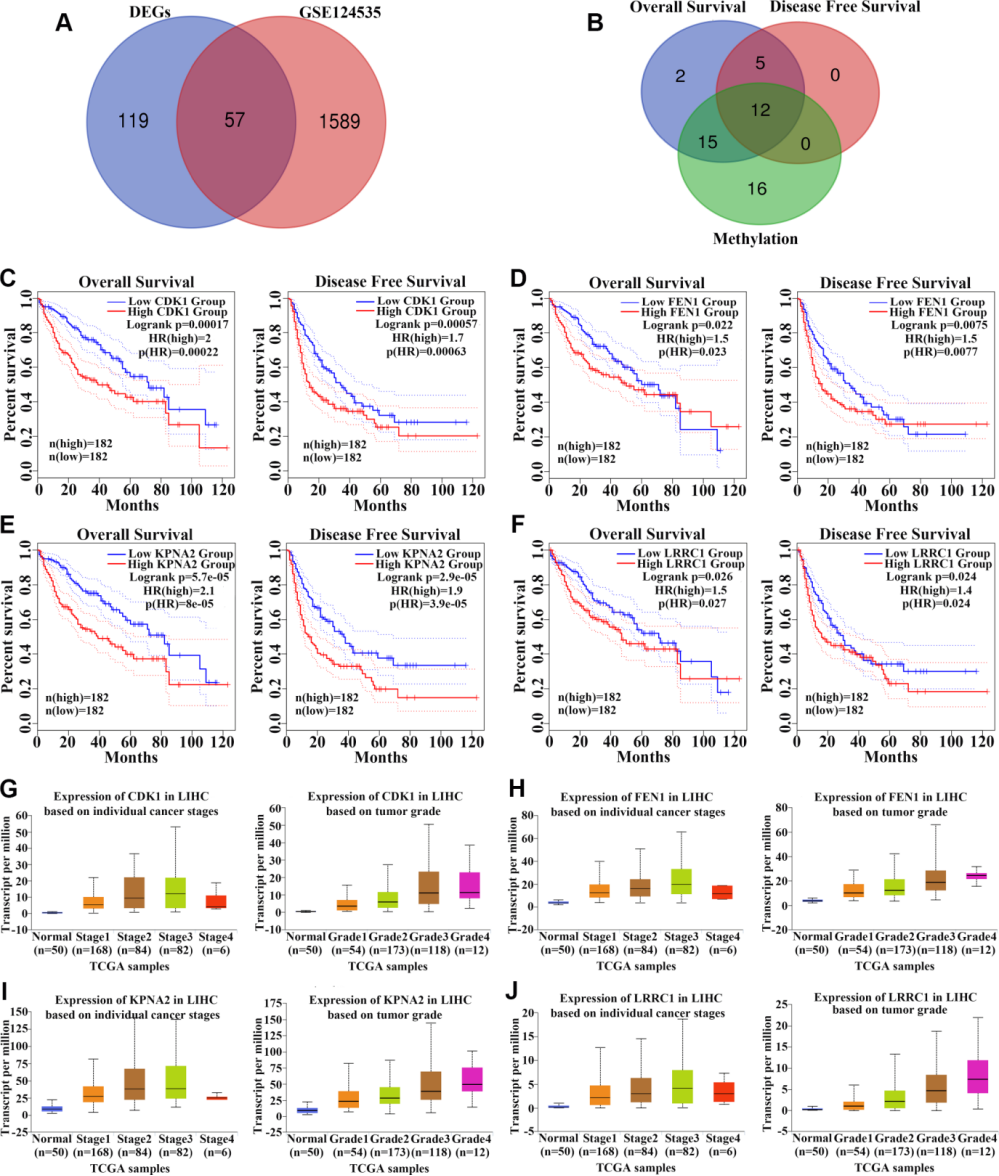
**Identification of 12 upregulated hub genes among HCC datasets.** (**A**) Among the 176 upregulated genes, 57 genes with higher protein levels in HCC tissues based on GSE124535 datasets were obtained. (**B**) We identified 12 upregulated hub genes by considering genes that were negatively associated with overall survival (OS) and disease-free survival (DFS) in HCC patients and genes that were hypomethylated. (**C**–**F**) Analysis of the association between *CDK1* (**C**), *FEN1* (**D**), *KPNA2* (**E**), and *LRRC1* (**F**) expression and OS/DFS among HCC patients in the TCGA cohort. (**G**–**J**) Analysis of the association between *CDK1* (**C**), *FEN1* (**D**), *KPNA2* (**E**), and *LRRC1* (**F**) expression and cancer stage/tumor grade among HCC patients in the TCGA cohort. *p-*values are shown in Supplementary Table 3.

**Figure 4:**
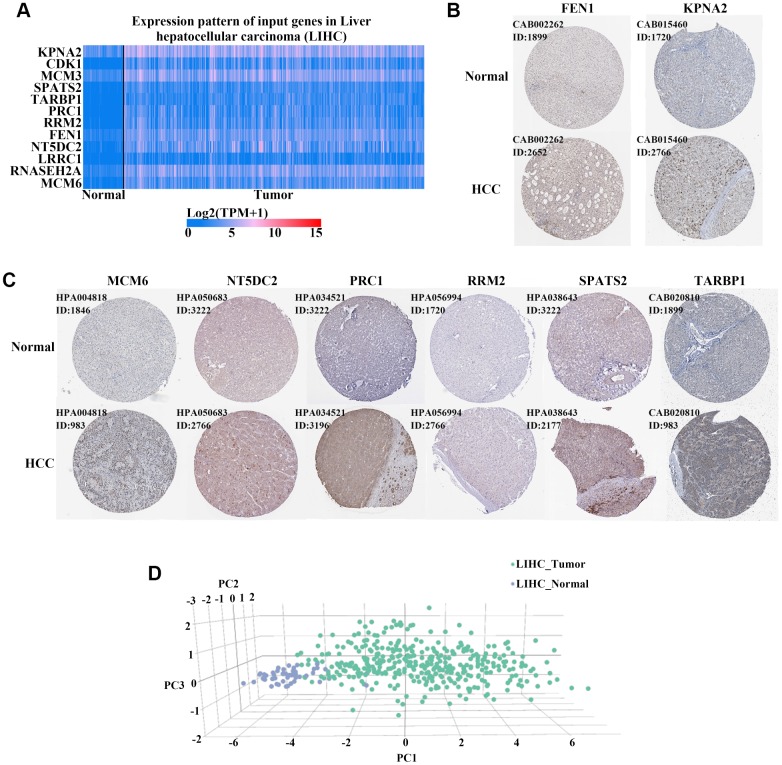
**Verification of the expression of 12 hub genes in HCC.** (**A**) Heatmaps of the levels of 12 hub genes comparing HCC and normal liver tissues in the TCGA cohort. Red and blue colors indicate higher and lower expression, respectively. (**B**–**C**) Eight hub genes were upregulated in HCC compared to expression in normal tissues based on immunohistochemical staining analysis of the Human Protein Atlas database. Antibody numbers and patient/healthy control ID numbers were annotated. (**D**) Three-dimensional (3D) principle component analysis (PCA) score plot showing that HCC patients can be effectively distinguished from healthy controls based on the expression of these 12 genes.

Among the 176 upregulated genes, we screened 57 (Supplementary Table 2) that were associated with higher protein levels in HCC tissues based on GSE124535 datasets (Figure 3A). We finally obtained 12 upregulated genes (*KPNA2*, *CDK1*, *MCM3*, *SPATS2*, *TARBP1*, *PRC1*, *RRM2*, *FEN1*, *NT5DC2*, *LRRC1*, *MCM6*, and *RNASEH2A*) by selecting overlapping genes between the hypomethylation set and those for which expression was negatively associated with overall survival (OS) and disease free survival (DFS) in HCC patients (Figure 3B). Levels of all 12 hub genes were negatively correlated with OS and DFS for HCC patients from the TCGA cohort (Figure 3C–3F, Supplementary Figure 1). In addition, the expression of these 12 genes was associated with individual cancer stages and tumor grade (Figure 3G–3J, Supplementary Figure 2). As shown in Figure 4A, mRNA levels of these 12 hub genes were higher in cancer than in normal liver tissues based on the TCGA cohort. Each column represents a sample and each row represents a gene; the color indicates the expression levels.

**Figure 5:**
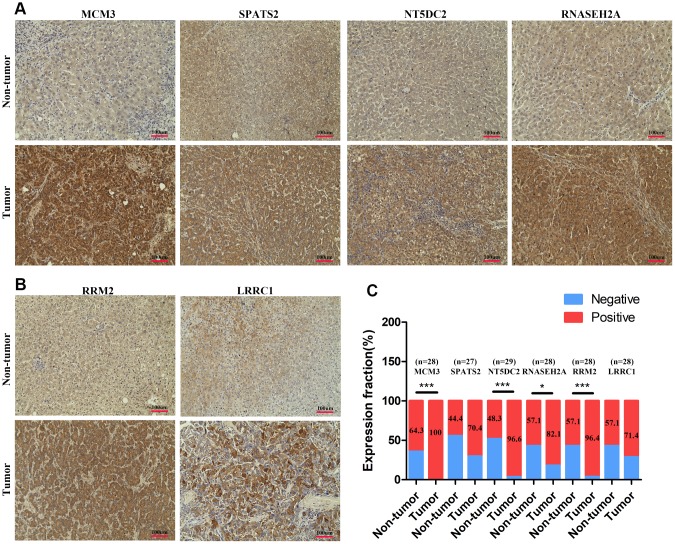
**The expression levels of MCM3, SPATS2, NT5DC2, RNASEH2A, LRRC1, and RRM2 in HCC tissues.** (**A**–**B**) Immunohistochemical staining analysed expression levels of MCM3, SPATS2, NT5DC2, RNASEH2A, LRRC1, and RRM2 in HCC and non-tumor tissues. (**C**) Positive expression percentage of the six genes in HCC and non-tumor tissues was showed. Fewer than 30 samples due to de-fragmentation. *P < 0.05; **P < 0.01; ***P < 0.001.

**Figure 6:**
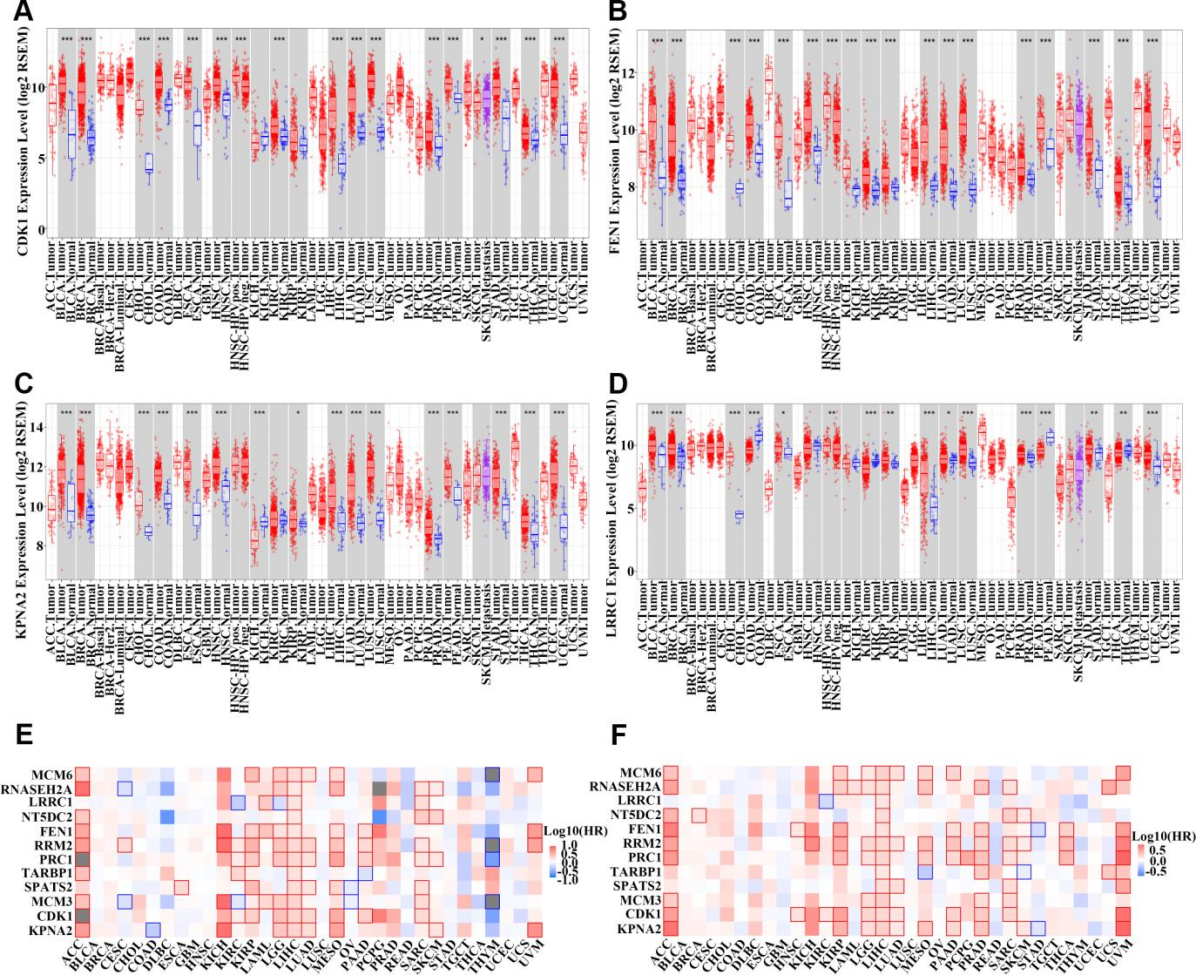
**Detection of the expression of 12 hub genes in other types of cancer.** (**A**–**D**) Boxplot of *CDK1* (**A**), *FEN1* (**B**), *KPNA2* (**C**), and *LRRC1* (**D**) expression in different types of cancer and normal tissues from the TCGA pan-cancer cohort. (**E**–**F**) Survival analysis examining the correlation between 12 hub genes and overall survival (OS) (**E**) or disease-free survival (DFS) (**F**) among different types of cancer patients in the TCGA cohort. Red wireframe indicates statistical differences. Red and blue colors show that gene expression was negatively and positively correlated with OS/DFS, respectively.

Next, we obtained protein expression data for eight genes from the Human Protein Atlas database. All eight genes were upregulated in HCC as compared to levels in normal tissues based on immunohistochemical staining analysis (Figure 4B–4C). A review of the literature found that six of the 12 upregulated genes (*KPNA2*, *CDK1*, *TARBP1*, *PRC1*, *FEN1*, and *MCM6*) were overexpressed in HCC tissue compared to levels in non-tumor tissue based on immunohistochemical staining [14–23]. We detected the expression of the other six genes (*MCM3*, *SPATS2*, *RRM2*, *NT5DC2*, *LRRC1*, and *RNASEH2A*) in 30 pairs of HCC and para-carcinoma tissue by immunohistochemical staining assays. MCM3 and RNASEH2A immunoreactivity was observed in both the cell nucleus and cytoplasm, whereas the other four proteins (SPATS2, RRM2, NT5DC2, and LRRC1) were all localized to the cytoplasm of HCC cells (Figure 5A–5B). The expression of four proteins (MCM3, RRM2, NT5DC2, and RNASEH2A) was significantly upregulated in HCC tissues compared to that in non-tumor tissues (Figure 5A–5C). The positive expression rate of SPATS2 and LRRC1 was higher in HCC tissues, but there was no statistical difference (*P* = 0.054 and *P* = 0.265 respectively; Figure 5C). Further increasing the number of samples might thus be required. In addition, based on the expression of these 12 genes, we could effectively distinguish HCC patients from healthy controls in the TCGA cohort by PCA analysis (Figure 4D). Furthermore, all of these genes, except *LRRC1*, were upregulated in most cancers, except the kidney chromophobe, in the TCGA pan-cancer cohort (Figure 6A–6D, Supplementary Figure 3). Survival analysis showed that the levels of these 12 genes were also associated with OS (Figure 6E) and DFS (Figure 6F) in a range of different cancer types.

### Functional analyses of upregulated hub genes

Studies have shown that KPNA2 can inhibit cell apoptosis and promote cell proliferation, migration, and invasion in HCC [24–26]. CDK1 can increase cellular viability and promote proliferation in HCC cell lines [15, 27]. Further, PRC1 can promote cell proliferation, migration, and invasion, promote tumor growth and metastasis, increase chemoresistance, and inhibit apoptosis in HCC [17–20]. It was also reported that RRM2 promotes HCC cell proliferation, inhibits apoptosis *in vitro*, and promotes tumor growth *in vivo* [28, 29]. FEN1 promotes HCC cell migration, invasion *in vitro* and promotes tumor growth and lung metastasis *in vivo* [21]. Meanwhile, LRRC1 enhances HCC cell proliferation *in vitro* and promotes tumor growth *in vivo* [30]. It was also reported that MCM6 increases the proliferative and migratory/invasive capability of HCC cells *in vitro*, in addition to increasing the tumor volume, weight, and the number of pulmonary metastases *in vivo* [22, 23].

**Figure 7:**
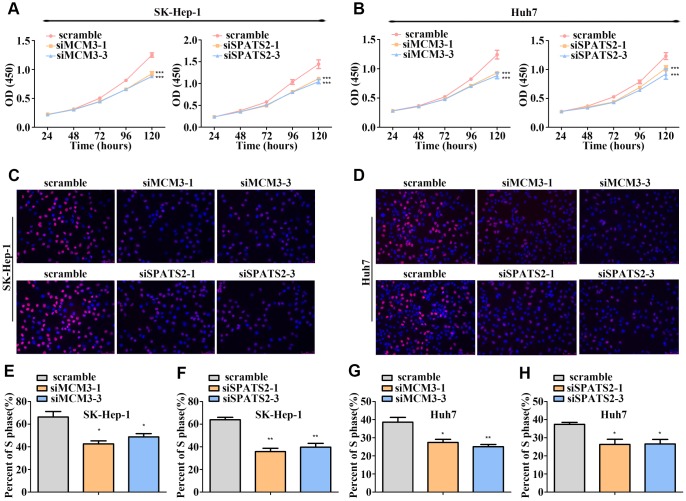
**MCM3 and SPATS2 promotes HCC cell proliferation.** (**A**–**B**) Proliferation of HCC cells with MCM3 or SPATS2 knockdown according to CCK-8 analysis. (**C**–**D**) EdU assays showing the proportion of S-phase cell after downregulating the expression of MCM3 or SPATS2. Nuclei of S-phase cells were pink. (**E**–**H**) Statistical analysis of EdU incorporation. *P < 0.05; **P < 0.01; ***P < 0.001.

**Figure 8:**
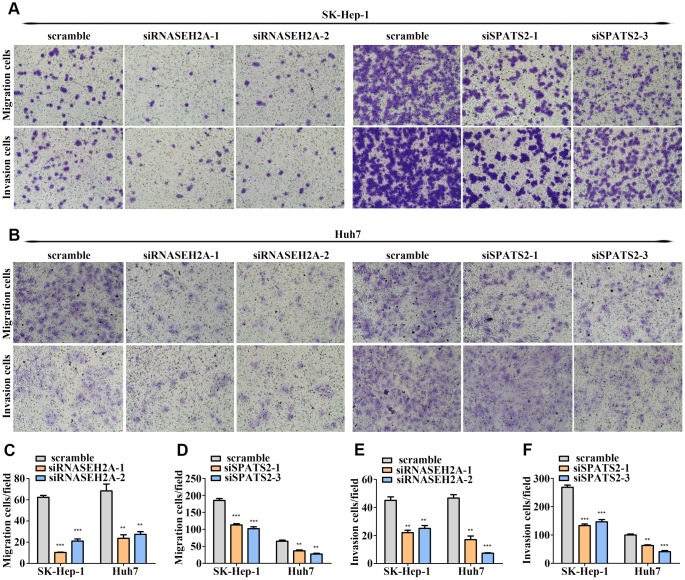
**RNASEH2A and SPATS2 promotes HCC cell migration and invasion.** (**A**–**B**) HCC cell migration and invasion were detected after downregulating the expression of RNASEH2A or SPATS2 by Transwell and Boyden assays. (**C**–**F**) Statistical analysis of Transwell and Boyden assay results. *P < 0.05; **P < 0.01; ***P < 0.001.

To examine the biological functions of MCM3, SPATS2, TARBP1, NT5DC2, and RNASEH2A in HCC, we transfected Huh7 and SK-Hep-1 cells with siRNA targeting these five genes. qRT-PCR and western blot assays verified the interference efficiency (Supplementary Figure 4A–4C). We found that silencing MCM3 expression suppressed Huh7 and SK-Hep-1 cell proliferation according to CCK-8 assays and reduced the percentage of S-phase cells according to EdU-incorporation assays (Figure 7) but had no effect on the migration and invasion of HCC cells (Supplementary Figure 6A–6C and Supplementary Figure 6E). SPATS2 knockdown suppressed the proliferation, migration, and invasion of Huh7 and SK-Hep-1 cells (Figure 7, Figure 8A–8B, Figure 8D, and Figure 8F). Silencing RNASEH2A expression decreased the migratory and invasive capability but did not affect the proliferative capacity of Huh7 and SK-Hep-1 cells (Figure 8A–8C, Figure 8E, and Supplementary Figure 5). Both NT5DC2 and TARBP1 knockdown did not affect HCC cell proliferation, migration, and invasion (Supplementary Figures 5 and 6). The expression of NT5DC2 could not be knocked down in SK-Hep-1 cells, and thus, we only performed functional analysis of this marker using Huh7 cells.

### Methylation and gene copy number analyses of upregulated hub genes

**Figure 9:**
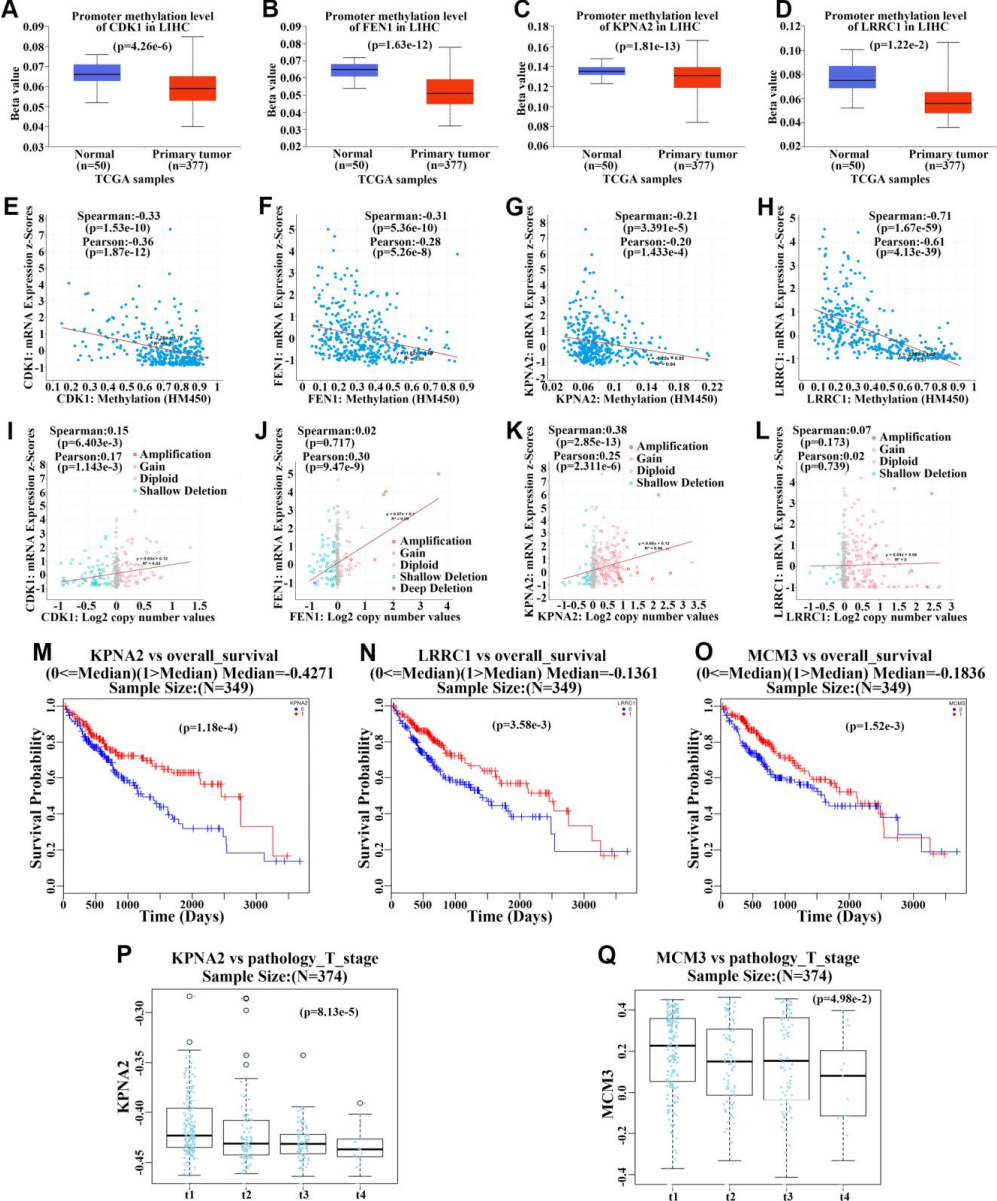
**Methylation and gene copy number analyses of upregulated hub genes.** (**A**–**D**) Methylation levels of *CDK1* (**A**), *FEN1* (**B**), *KPNA2* (**C**), and *LRRC1* (**D**) in primary hepatocellular carcinoma (HCC) tumors and normal tissues from the TCGA cohort. (**E**–**H**) Correlation analysis of methylation levels of *CDK1* (**E**), *FEN1* (**F**), *KPNA2* (**G**), and *LRRC1* (**H**) and their mRNA expression in HCC based on the TCGA cohort. (**I**–**L**) Correlation analysis of gene copy numbers of *CDK1* (**I**), *FEN1* (**J**), *KPNA2* (**K**), and *LRRC1* (**L**) and their mRNA expression in HCC based on the TCGA cohort. (**M**–**O**) Survival analysis of the correlation between methylation levels of *KPNA2* (**M**), *LRRC1* (**N**), and *MCM3* (**O**) and overall survival (OS) in HCC patients from the TCGA cohort. (**P**–**Q**) Analysis of the association between *KPNA2* (**P**) and *MCM3* (**Q**) methylation and pathologic T stage in HCC patients of the TCGA cohort.

We further determined the methylation status of the aforementioned 12 upregulated genes in the TCGA cohort and analyzed the correlation between mRNA expression and DNA methylation levels. The 12 genes had lower methylation levels in primary HCC compared to those in normal liver tissues (Figure 9A–9D, Supplementary Figure 7A–7D, and Supplementary Figure 7I–7L). Additionally, DNA methylation levels of all hub genes were negatively associated with mRNA expression (Figure 9E–9H, Supplementary Figure 7E–7H, and Supplementary Figure 7M–7P), suggesting that DNA methylation might regulate the mRNA expression of these genes. Further, the associations between copy number and the mRNA expression levels of the 12 genes were also tested. The mRNA levels of seven genes including *CDK1*, *FEN1*, *KPNA2*, *MCM3*, *RRM2*, *SPATS2*, and *TARBP1* were found to be positively related to copy number (Figure 9I–9L, Supplementary Figure 8), indicating that gene copy number might also contribute to the upregulation of these genes. Additionally, the methylation level of *KPNA2*, *LRRC1*, and *MCM3* was positively associated with OS for HCC patients (Figure 9M–9O), and the methylation level of *KPNA2* and *MCM3* was negatively associated with pathologic T stage for this cohort (Figure 9P–9Q). This suggested that the methylation status of these three genes is related to clinical features among HCC patients.

### Correlation between hub gene levels and immune cell infiltration

**Figure 10:**
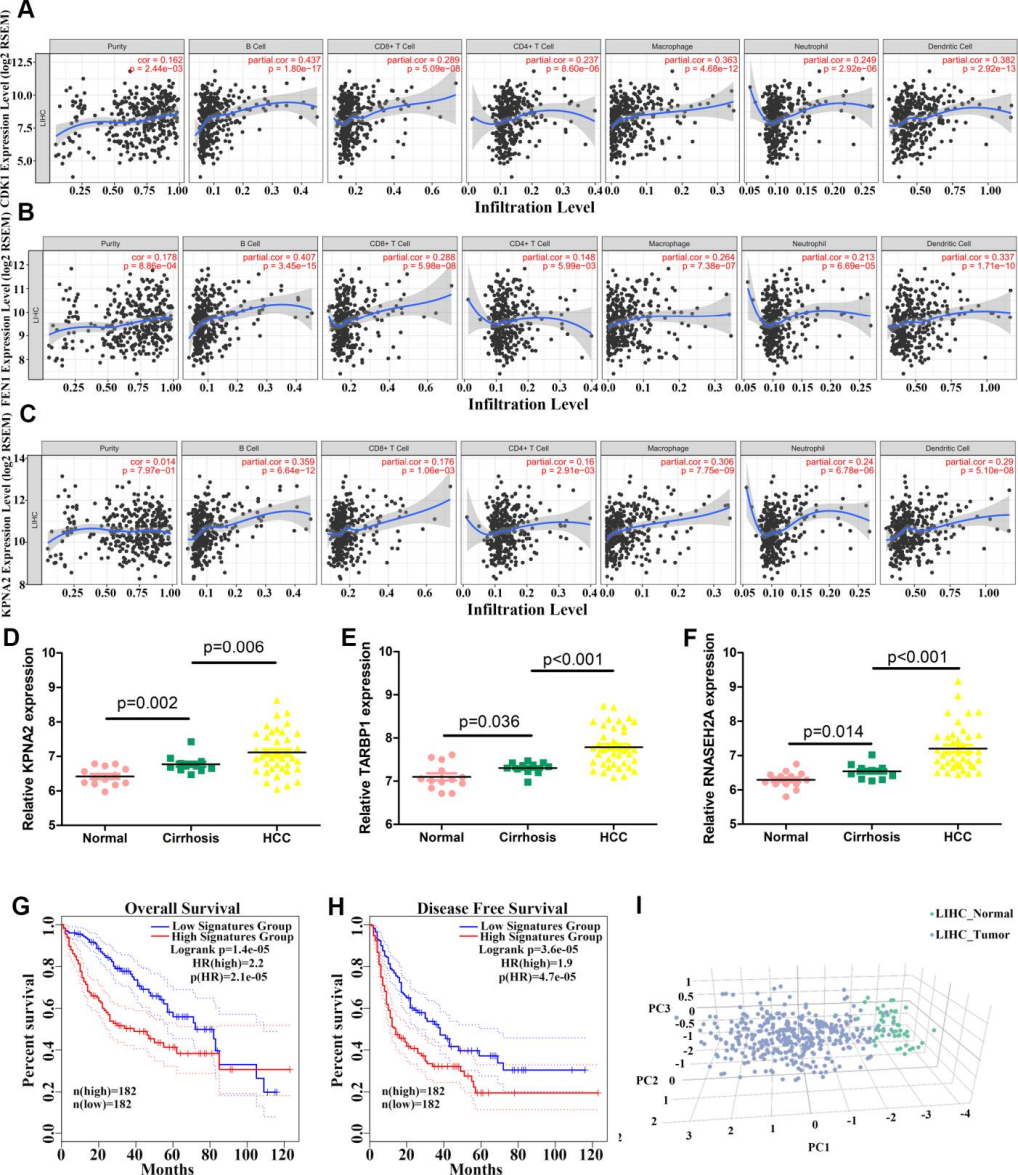
**Correlation between levels of hub genes and immune cell infiltration and identification of three hub genes.** (**A**–**C**) Correlation between *CDK1* (**A**), *FEN1* (**B**), and *KPNA2* (**C**) levels and immune cell infiltration in hepatocellular carcinoma (HCC) tissues. Each dot represents a sample in the TCGA cohort. (**D**–**F**) *KPNA2* (**D**), *TARBP1* (**E**), and *RNASEH2A* (**F**) mRNA levels in normal, cirrhosis, and HCC samples from GSE89377. (**G**–**H**) Analysis of the correlation between three-hub gene expression signatures and overall survival (OS) (**G**) or disease-free survival (DFS) (**H**) for HCC patients of the TCGA cohort. (**I**) HCC patients could be effectively distinguished from healthy controls by principle component analysis (PCA) based on expression of the three genes.

We then performed an interrelation analysis comparing infiltrating immune cells in HCC tissues and the expression of upregulated genes, except *LRRC1*, using Timer software. Among these 11 genes, the expression levels of all but *TARBP1* were positively related to the infiltration levels of B cells, CD8^+^ T cells, CD4^+^ T cells, macrophages, neutrophils, and dendritic cells in HCC tissues, suggesting that higher expression levels of these 10 genes indicate an advantage for cancer immunotherapy (Figure 10A–10C and Supplementary Figure 9).

### A combination of the three upregulated hub genes can provide diagnostic and prognostic value for HCC patients

We finally selected three upregulated hub genes (*KPNA2*, *TARBP1*, and *RNASEH2A*) for which expression was systematically increased from normal liver to cirrhosis and HCC tissues based on the GSE89377 dataset (Figure 10D–10F). Combined expression signatures of these three genes were negatively related to OS and DFS for HCC patients in the TCGA cohort (Figure 10G–10H). The signature score was calculated based on the mean log_2_(TPM+1) value of each gene. Further, we could effectively and accurately distinguish HCC patients from healthy controls by PCA analysis based on the three-gene expression signature (Figure 10I).

## DISCUSSION

HCC remains a dominant cause of cancer-related death despite dramatic improvements in its treatment. Here, we screened DEGs in HCC tissues and investigated these genes in-depth based on expression levels, survival, methylation status, DNA copy number, and immune cell infiltration. We finally identified three hub genes that could provide comprehensive diagnostic and prognostic value for HCC patients.

GO enrichment and functional pathway analyses of 176 upregulated genes showed that these genes were mainly involved in cell cycle, the Aurora B pathway, positive regulation of cell cycle, cell cycle G1/S phase transition, and the PLK1 pathway. Aurora B is a crucial regulator of accurate mitosis and its abnormal expression is associated with cancer progression [31, 32]. The PLK1 pathway also participates in the regulation of cell mitosis and acts as an oncogenic factor to promote cancer development [33, 34]. PPI network analysis demonstrated the most important regulatory functions of the upregulated genes, including sister chromatid cohesion, mitotic prometaphase, M phase, cell cycle, and DNA conformational changes. This was consistent with GO analysis results. These results indicate that the upregulated genes are mainly involved in tumor growth modulation, suggesting their relevance to HCC pathogenesis and progression.

Combining survival and DNA methylation analyses, we obtained 12 upregulated hub genes. The majority of these were previously proven to play important roles in cancer progression [16, 30, 35–42], indicating the consistency of our data with other research reports. Their levels were also positively associated with cancer stages and tumor grade, suggesting significant roles in the development of HCC. Furthermore, we showed that all 12 genes could predict HCC patient prognosis and that a combination of them could serve as a biomarker to accurately distinguish patients from healthy controls. Indeed, we provide foundational data suggesting that all of these genes could serve as promising prognostic indicators and therapeutic targets.

The observed hypomethylation of the 12 genes and the negative correlation between methylation status and mRNA levels in HCC indicate that DNA hypomethylation could account for such abnormal expression patterns. Nevertheless, the copy numbers of only seven genes (*CDK1*, *FEN1*, *KPNA2*, *MCM3*, *RRM2*, *SPATS2*, and *TARBP1*) were found to be positively associated with mRNA levels. This indicates that DNA methylation and copy number might coordinately modulate the expression of these genes.

Cancer immunotherapy, and especially multiple immune checkpoint inhibitors, has received increasing attention and has become a promising treatment strategy in recent years [43, 44]. The levels of all hub genes identified in this study, except *TARBP1* and *LRRC1*, were able to predict immune cell infiltration in HCC tissues. Reports have shown that the clinical efficacy of checkpoint inhibitors is significantly dependent on the number of pre-existing tumor-infiltrating immune cells [45, 46]. Our data suggested that higher expression levels of these 10 genes could indicate an advantage for checkpoint inhibitor therapy, potentially providing guidance for cancer immunotherapy. To better screen prognostic and diagnostic markers, we finally identified three hub genes (*KPNA2*, *TARBP1*, and *RNASEH2A*) for which expression could collectively and precisely predict patient prognosis. The combination of these three genes will be more meaningful and convenient for clinical applications than the 12 genes.

In conclusion, by integrating bioinformatic analyses, we obtained a combination of several hub genes with potential clinical significance for HCC. More work is required to better reveal their functions and the underlying mechanisms with respect to HCC progression, as well as their potential applications for disease diagnosis and prognosis.

## MATERIALS AND METHODS

### Microarray data

The mRNA expression data, methylation data, and corresponding clinical information for HCC patients were obtained from The Cancer Genome Atlas (TCGA; https://tcga-data.nci.nih.gov/tcga/) cohort. The GEPIA2 website was used to screen DEGs between HCC samples and normal tissue and to visualize the expression patterns and chromosomal locations of the upregulated genes.

Additionally, four microarray datasets (GSE124535, GSE112790, GSE121248, and GSE89377) were downloaded from the Gene Expression Omnibus (GEO; http://www.ncbi.nlm.nih.gov/geo) databases. GSE124535 data are based on GPL20795 platforms and include mRNA and protein expression information from 35 paired HCC and non-tumor tissues. GSE112790 data are based on GPL570 platforms and include mRNA expression information from 183 HCC patients and 15 adjacent liver tissues. GSE121248 data are based on GPL570 platforms and include mRNA expression information from 70 HCC and 37 adjacent normal tissues. GSE89377 data are based on GPL6947 platforms and include mRNA expression information from 107 samples covering different stages of HCC development. GEO2R was used to screen DEGs between HCC samples and non-tumor controls. We used the criteria of a *p*-value < 0.01 and [log_2_FC] > 1 to define DEGs, as well as an online Venn diagram tool (http://bioinfogp.cnb.csic.es/tools/venny/) to screen overlapping DEGs.

### Functional enrichment analysis, establishment of protein-protein interaction (PPI) network, and modular analysis of upregulated genes

Functional enrichment analysis was performed using Metascape (http://metascape.org/gp/index.html#/main/step1). Specifically, we submitted our 176 upregulated genes into this platform. Gene Ontology (GO) terms for biological process, cellular component, and molecular function categories, as well as Kyoto Encyclopedia of Genes and Genomes pathways, were found to be enriched and a *p*-value < 0.05 was considered statistically significant. The most enriched term within a cluster was represented.

Metascape can be used to automatically analyze the biological significance of a large number of genes. We use Metascape and Cytoscape software to construct the PPI network comprising the 176 upregulated genes. We identified all significantly enriched terms, which were then hierarchically clustered into a tree based on Kappa-statistical similarities among their gene memberships. Next, a 0.3 kappa score was applied as the threshold to divide the tree into term clusters, and a subset of representative terms were selected from this cluster and converted into a network layout. More specifically, each term was represented by a circle node, where its size was proportional to the number of input genes that fell into that term, and its color represented its cluster identity. Terms with a similarity score > 0.3 were linked by an edge (the thickness of the edge represents the similarity score) and the network was visualized with Cytoscape (v3.1.2) (http://www.cytoscape.org/) with a “force-directed” layout and with edges bundled for clarity. We used Molecular Complex Detection (MCODE), a plug-in in Cytoscape, to filter the modules from the PPI network and to obtain the most important module based on the MCODE score and node number.

### Survival analysis

The association between the expression levels of DEGs and OS or DFS was analyzed using the Kaplan–Meier survival method based on the TCGA HCC cohort. Cancer samples were divided into two groups based on the expression of genes to plot survival curves. A *p*-value < 0.05 was regarded as statistically significant.

### Validation of the 12 upregulated genes

An expression heatmap of the 12 genes in HCC and normal liver tissues based on the TCGA cohort was produced using UALCAN software (http://ualcan.path.uab.edu/index.html). To detect translational levels of the 12 genes, we obtained immunohistochemistry sections of HCC and normal tissues from the Human Protein Atlas database (https://www.proteinatlas.org/). Furthermore, we constructed expression box plots for the 12 genes in different types of tumors and normal tissues based on data from the TCGA pan-cancer cohort using Timer software (https://cistrome.shinyapps.io/timer/).

### Methylation and gene copy number analyses

We compared the methylation levels of 12 genes between normal and primary tumor tissues based on information from the TCGA HCC cohort, which included human cancer methylation data from microarray and sequencing technology. We also examined the association between the expression levels of the 12 genes and their DNA methylation patterns or copy numbers using cBioPortal software (http://www.cbioportal.org/).

### Correlation between gene expression and immune cell infiltration

We used Timer software (https://cistrome.shinyapps.io/timer/), which includes different types of cancer samples accessible in the TCGA cohort, to examine the correlation between expression of the 12 genes and tumor-infiltrating immune cells (TIICs; B cells, CD4^+^ T cells, CD8^+^ T cells, neutrophils, macrophages, and dendritic cells). Timer applies a deconvolution method to infer the abundance of TIICs from gene expression profiles. In brief, the informative immune signature genes that are negatively associated with tumor purity (percentage of malignant cells in a tumor tissue) for each tumor type were selected. Then, constrained least squares fitting on the immune signature genes was used to infer the abundance of TIICs [47, 48].

### Principal component analysis (PCA)

We performed PCA using GEPIA2 websites to distinguish HCC patients from healthy controls based on levels of the upregulated genes. PCA is capable of reducing the dimensionality of redundant and noisy information from complex massive datasets. We transformed the original variables into three new orthogonal variables called principal components (PCs). A PC score plot was obtained to represent clear clustering of the target points.

### siRNA transfection

siRNA was purchased from RiboBio (Guangzhou, China) and was transfected into HCC cells using Lipofectamine RNAiMAX (Invitrogen) at a working concentration of 100 nM according to manufacturer instructions.

### Cell culture, quantitative reverse transcription polymerase chain reaction (qRT-PCR), Western blotting, Cell Counting Kit (CCK)-8 assay, 5-ethynyl-2′-deoxyuridine (EdU)-incorporation assay, transwell-migration, and Boyden-invasion assays

The details of these assays were described previously [49]. Primer sequences are listed in Supplementary Table 4. Antibodies are listed in Supplementary Table 5.

### Immunohistochemical staining

Immunohistochemical staining of the HCC tissue microarray (Chaoxing Biotechnology, Shanghai, China) was carried out following the manufacturer’s protocol. The score standard for the intensity of staining was as follows: 0, negative; 1, weak; 2, medium; 3, strong. The extent of staining was scored as: 0, 0%; 1, 1–25%; 2, 26–50%; 3, 51–75%; 4, 76–100%. Total scores of 2 or lower were defined as the negative group, whereas total scores of 3 or higher were defined as the positive group. Antibodies are listed in Supplementary Table 5.

### Statistical analysis

We used SPSS 16.0 software (Chicago, IL, USA) to perform data analysis. A Student’s *t* test was utilized to assess significance of data from two groups, and one-way analysis of variance (ANOVA) followed by Dunnett’s multiple comparison was performed to evaluate differences between multiple groups. Correlation analysis was undertaken using Pearson or Spearman tests. Overall and disease-free survival were evaluated using the Kaplan–Meier method. *P* < 0.05 was considered statistically significant.

## Supplementary Material

Supplementary Figures

Supplementary Tables

Supplementary Table 1

Supplementary Table 3
