# Supplementary material for: Identification of hub genes in hepatocellular carcinoma using integrated bioinformatic analysis
Source: Aging (Albany NY). 2020 Mar 26;12(6):5439–68. doi: 10.18632/aging.102969 (PMC7138582; doi:10.18632/aging.102969)
Supplement: Supplementary Figures [file aging-12-102969-s004..pdf]

SUPPLEMENTARY FIGURES

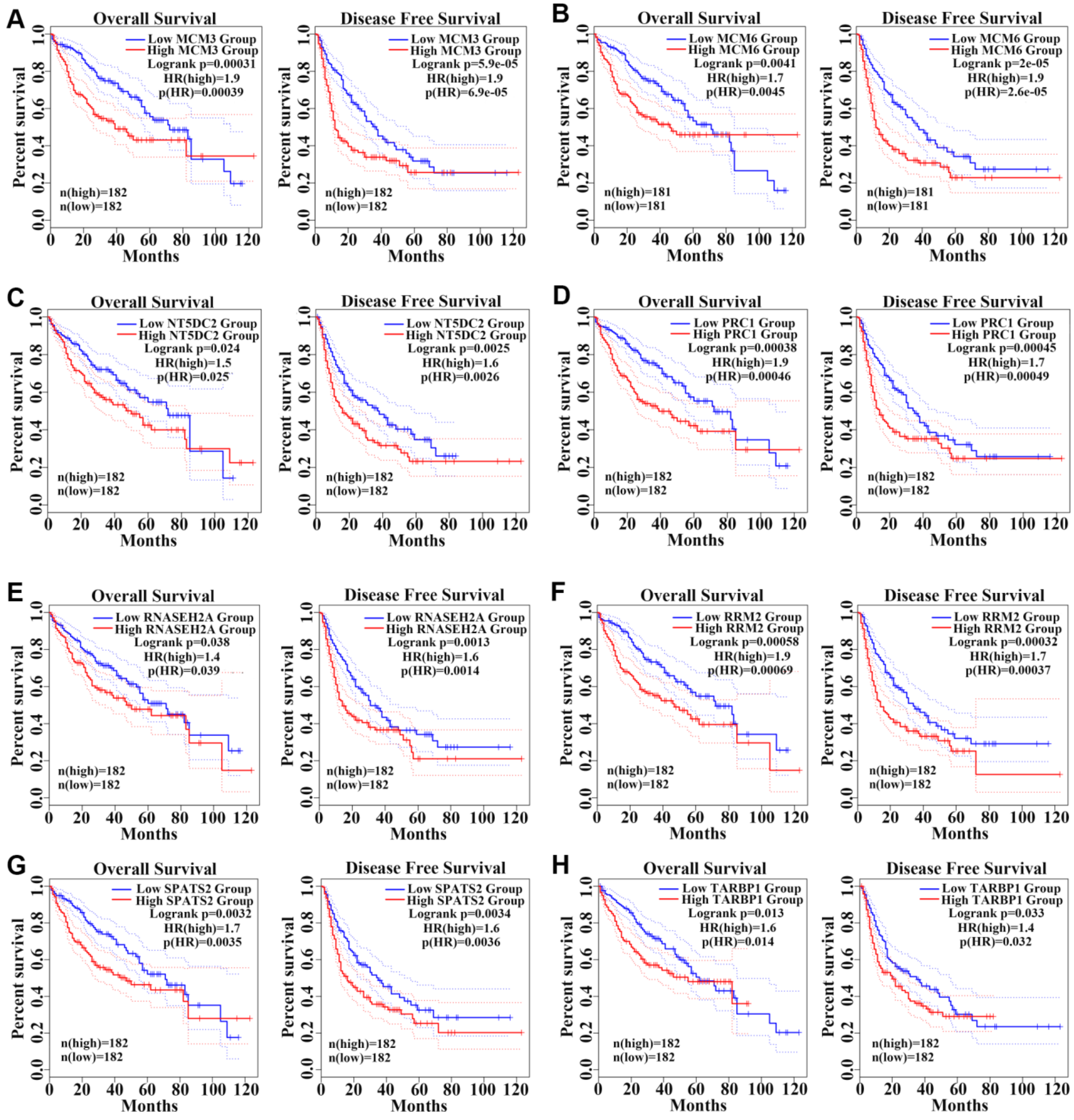

Supplementary Figure 1. Survival analysis based on upregulated hub genes. (A–H) Survival analysis of the association between *MCM3* (A), *MCM6* (B), *NT5DC2* (C), *PRC1* (D), *RNASEH2A* (E), *RRM2* (F), *SPATS2* (G), and *TARBP1* (H) expression and overall survival (OS) or disease-free survival (DFS) based on hepatocellular carcinoma (HCC) patients in the TCGA cohort.

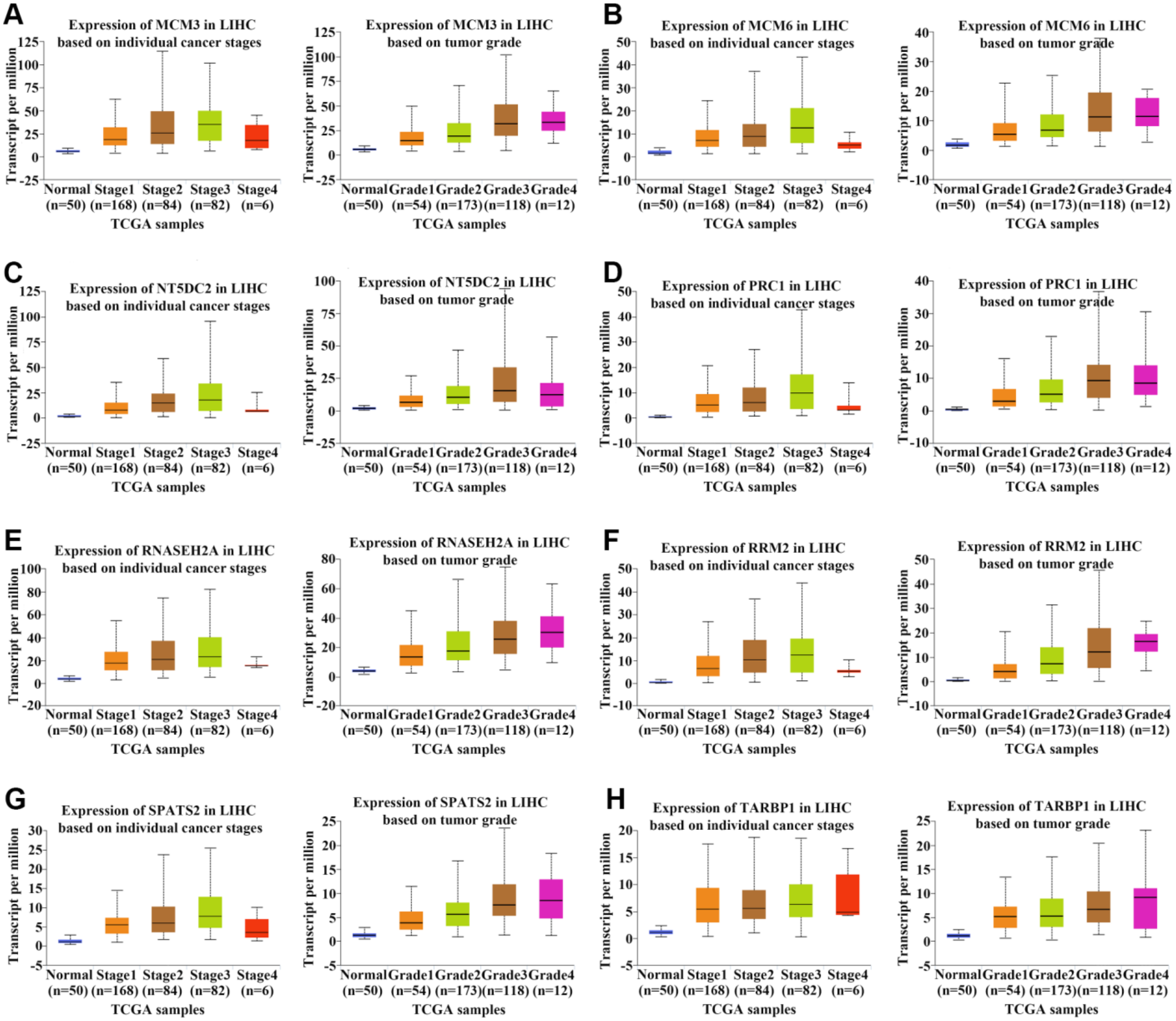

Supplementary Figure 2. Analysis of clinical features associated with upregulated hub genes. (A–H) Analysis of the association between *MCM3* (A), *MCM6* (B), *NT5DC2* (C), *PRC1* (D), *RNASEH2A* (E), *RRM2* (F), *SPATS2* (G), and *TARBP1* (H) expression and cancer stage/tumor grade based on hepatocellular carcinoma (HCC) patients in the TCGA cohort. *p*-values are shown in Supplementary Table 3.

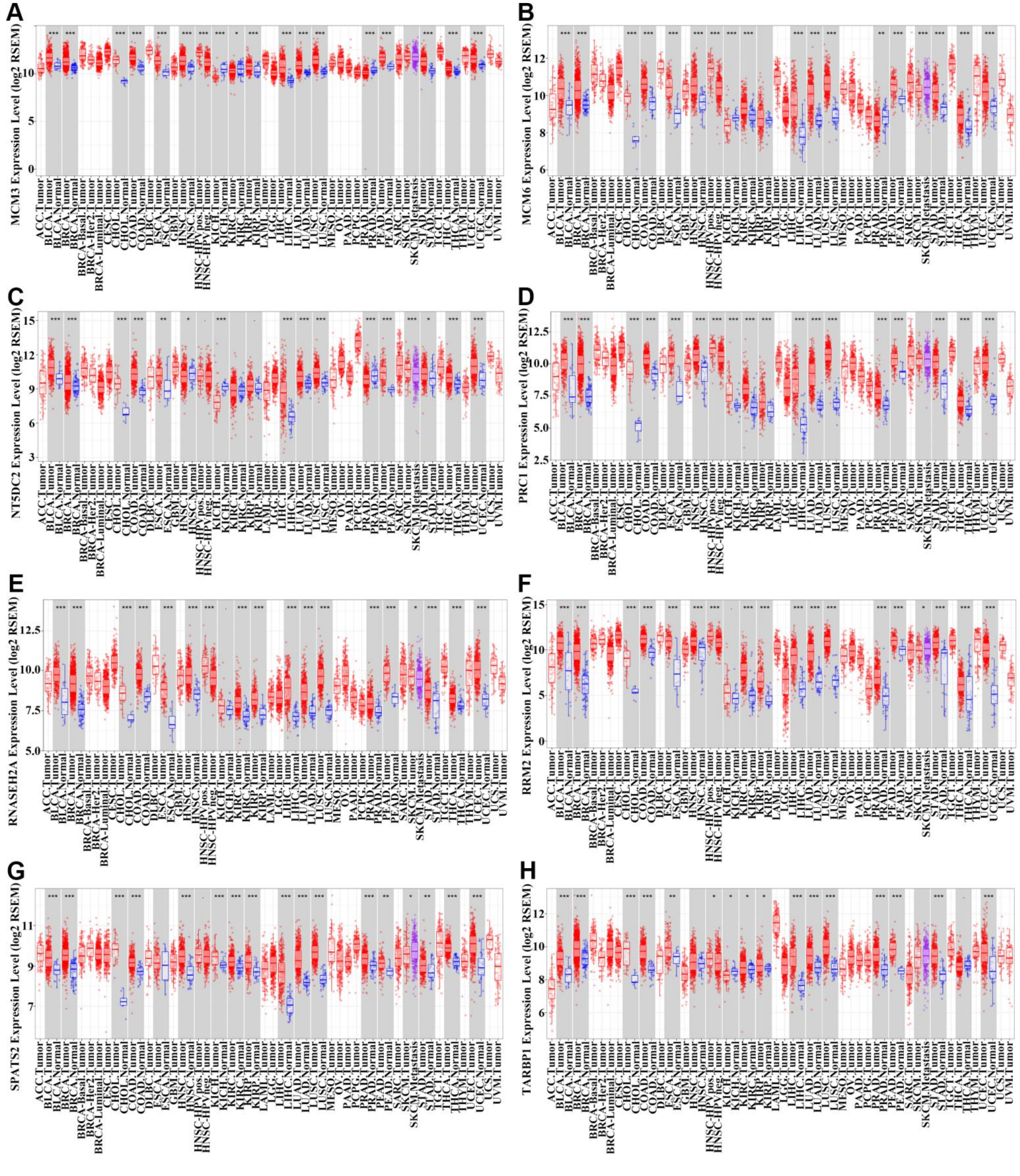

Supplementary Figure 3. Expression of upregulated hub genes in different types of cancer. (A–H) Expression of *MCM3* (A), *MCM6* (B), *NT5DC2* (C), *PRC1* (D), *RNASEH2A* (E), *RRM2* (F), *SPATS2* (G), and *TARBP1* (H) in different types of cancer and normal tissues from the TCGA pan-cancer cohort.

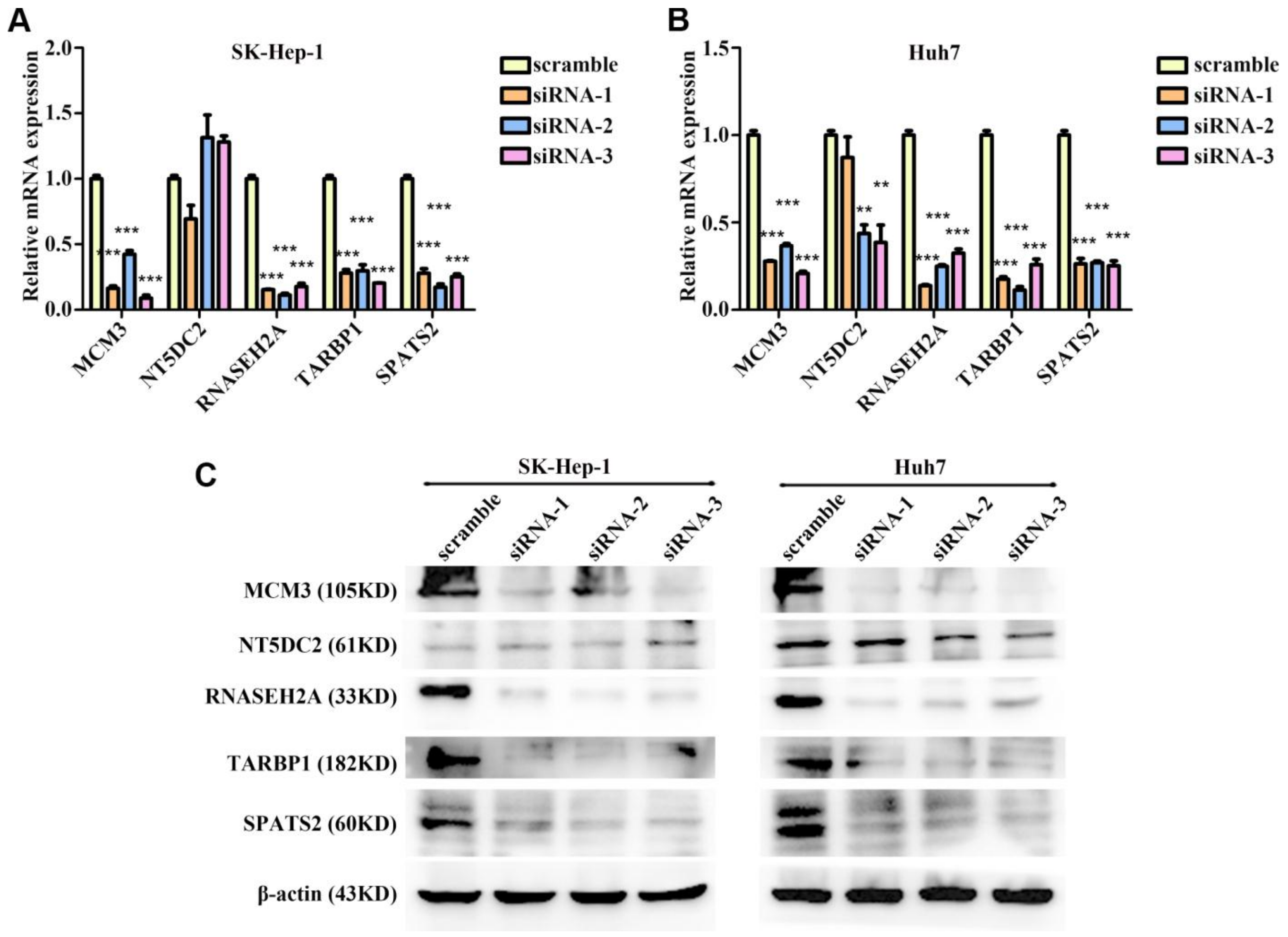

Supplementary Figure 4. Transfection efficiency verification. (A-C) Transfection efficiency of siRNA for the five genes in HCC cells according to qRT-PCR (A-B) and western blotting (C) assays. **P* < 0.05; ***P* < 0.01; ****P* < 0.001.

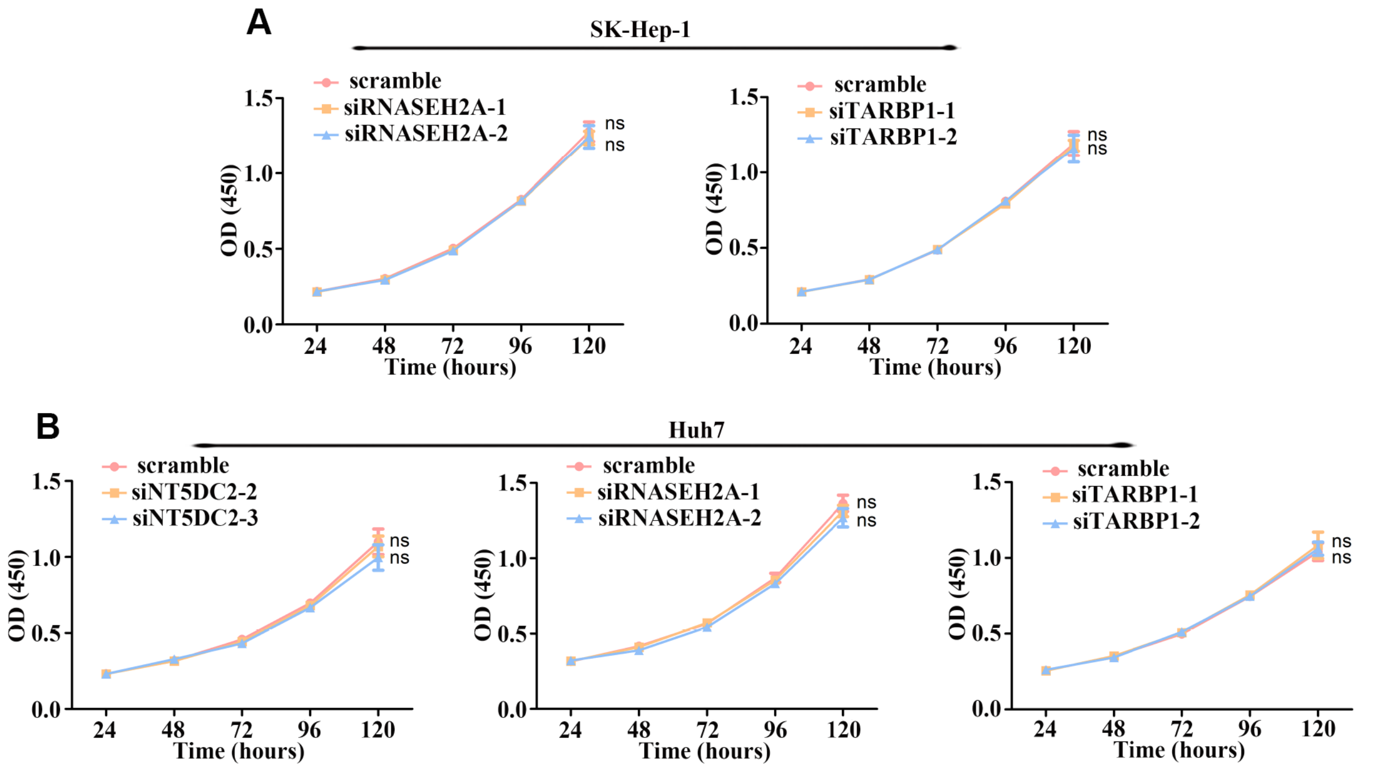

Supplementary Figure 5. RNASEH2A, TARBP1, and NT5DC2 does not affect HCC cell proliferation. (A-B) Proliferation of HCC cells with RNASEH2A, TARBP1, or NT5DC2 knockdown according to CCK-8 analysis. ns: no statistical differences.

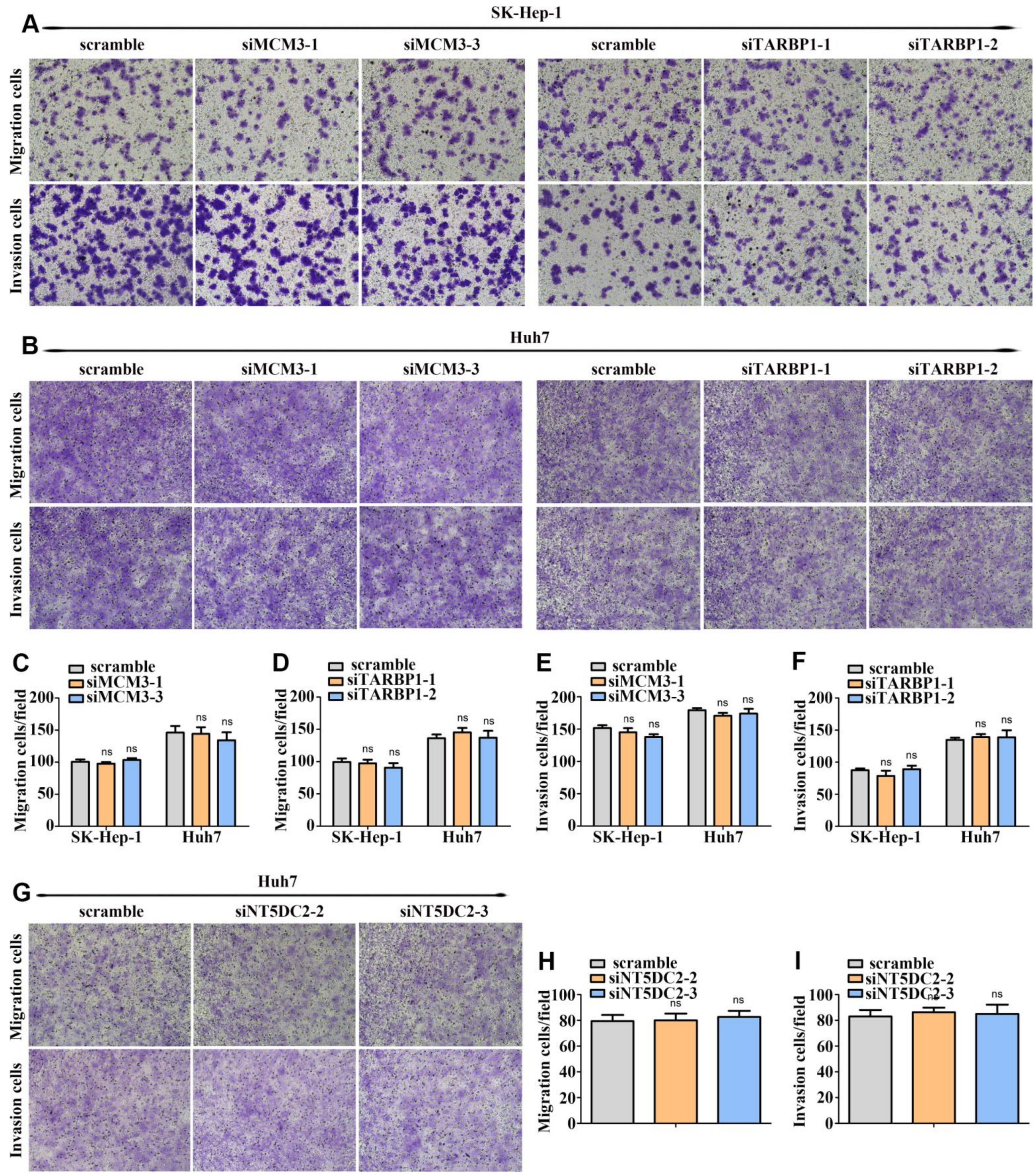

Supplementary Figure 6. MCM3, TARBP1, and NT5DC2 does not affect HCC cell migration and invasion. (A-B, and G) HCC cell migration and invasion were evaluated after downregulating the expression of MCM3, TARBP1, or NT5DC2 by Transwell and Boyden assays. (C-F, and H-I) Statistical analysis of Transwell and Boyden assay results. ns: no statistical differences.

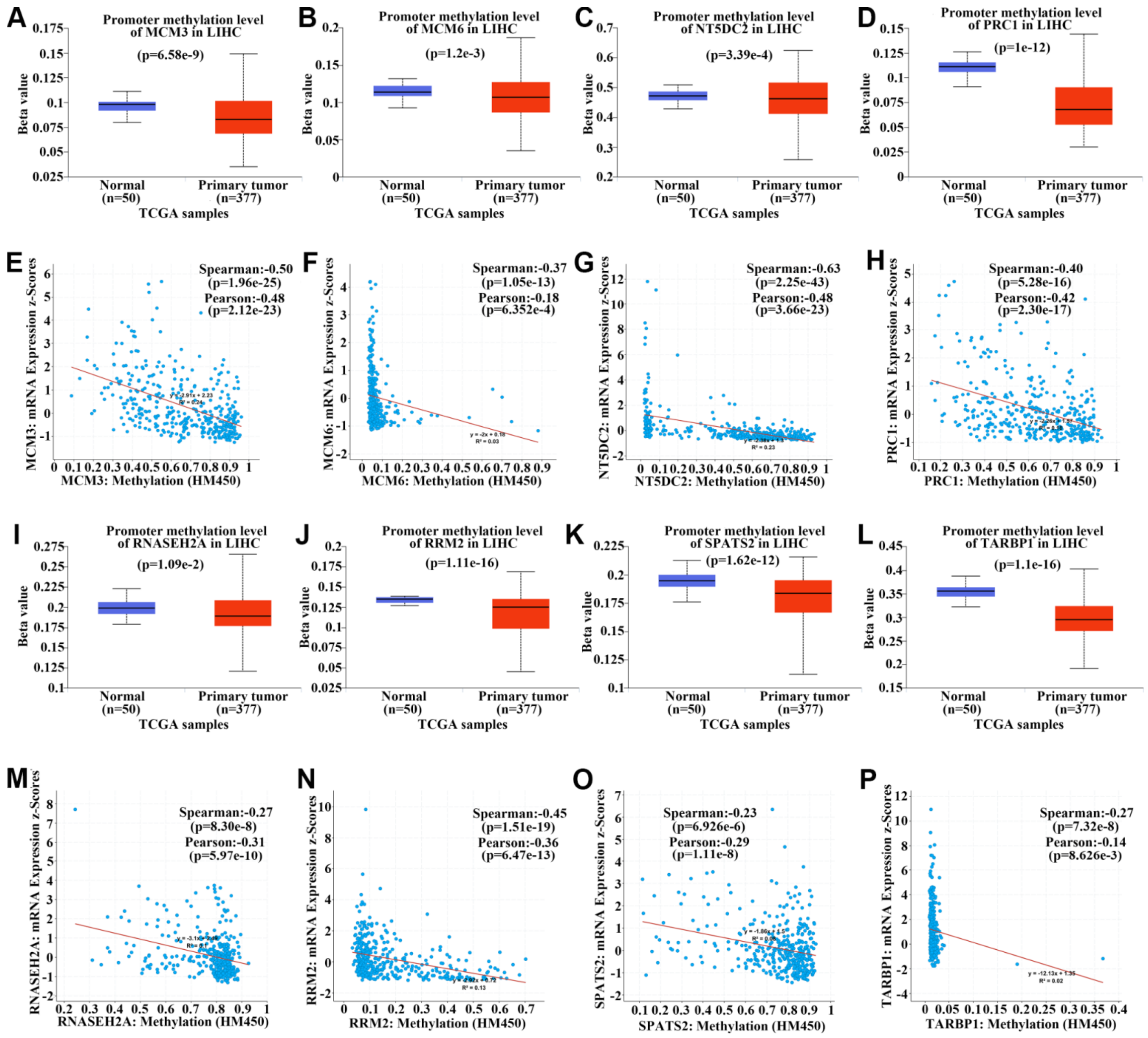

Supplementary Figure 7. Methylation analysis of upregulated hub genes. (A–D, I–L) Methylation levels of *MCM3* (A), *MCM6* (B), *NT5DC2* (C), *PRC1* (D), *RNASEH2A* (I), *RRM2* (J), *SPATS2* (K), and *TARBP1* (L) in primary hepatocellular carcinoma (HCC) tumors and normal tissues from the TCGA cohort. (E–H, M–P) Correlation analysis comparing methylation levels of *MCM3* (E), *MCM6* (F), *NT5DC2* (G), *PRC1* (H), *RNASEH2A* (M), *RRM2* (N), *SPATS2* (O), and *TARBP1* (P) with their mRNA expression in HCC based on the TCGA cohort.

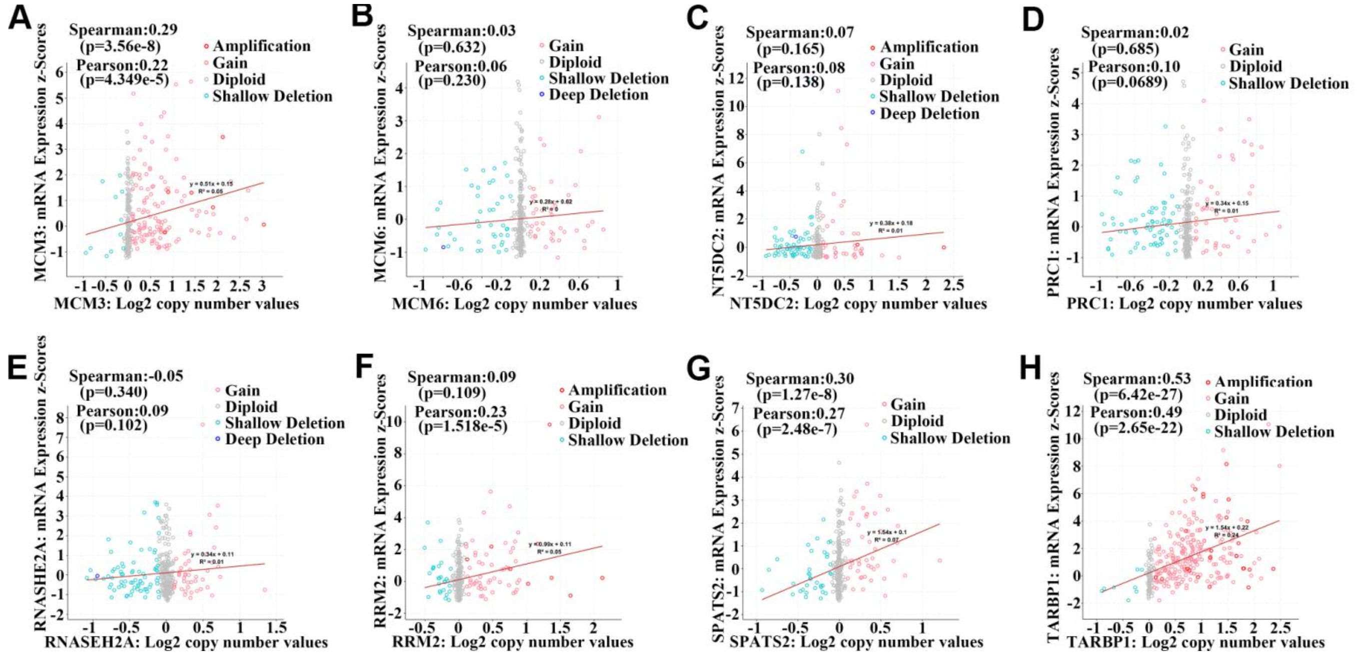

Supplementary Figure 8. Gene copy number analysis of upregulated hub genes. (A–H) Correlation analysis of gene copy numbers of *MCM3* (A), *MCM6* (B), *NT5DC2* (C), *PRC1* (D), *RNASEH2A* (E), *RRM2* (F), *SPATS2* (G), and *TARBP1* (H) and their mRNA expression levels in hepatocellular carcinoma (HCC) based on the TCGA cohort.

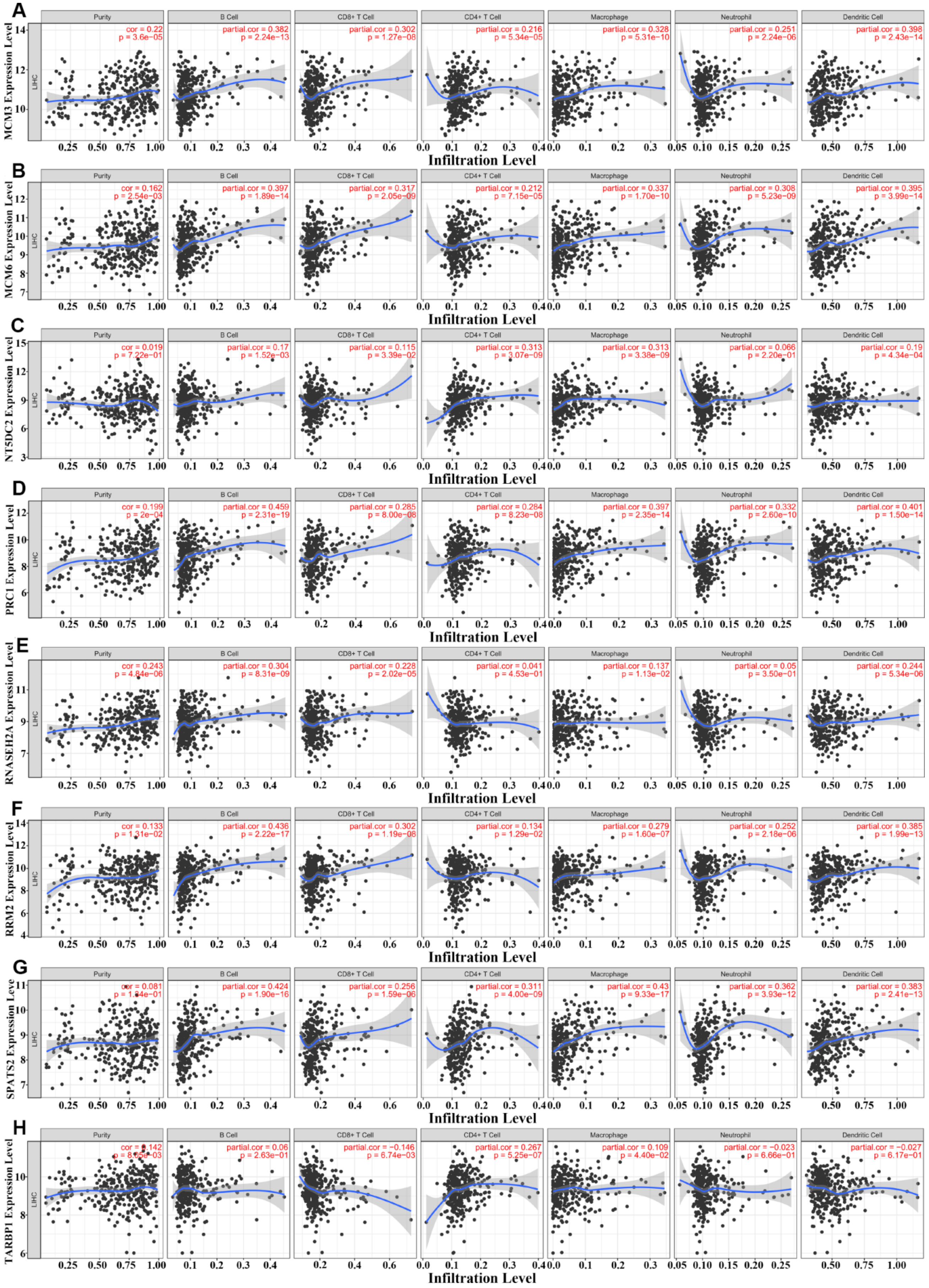

A
Purity
B Cell
CD8+ T Cell
CD4+ T Cell
Macrophage
Neutrophil
Dendritic Cell
LIHC
MCM3 Expression Level
cor = 0.22
p = 3.6e-05
partial.cor = 0.382
p = 2.24e-13
partial.cor = 0.302
p = 1.27e-08
partial.cor = 0.216
p = 5.34e-05
partial.cor = 0.328
p = 5.31e-10
partial.cor = 0.251
p = 2.24e-06
partial.cor = 0.398
p = 2.43e-14
Infiltration Level
B
MCM6 Expression Level
cor = 0.162
p = 2.54e-03
partial.cor = 0.397
p = 1.89e-14
partial.cor = 0.317
p = 2.05e-09
partial.cor = 0.212
p = 7.15e-05
partial.cor = 0.337
p = 1.70e-10
partial.cor = 0.308
p = 5.23e-09
partial.cor = 0.395
p = 3.99e-14
Infiltration Level
C
NT5DC2 Expression Level
cor = 0.019
p = 7.22e-01
partial.cor = 0.17
p = 1.52e-03
partial.cor = 0.115
p = 3.39e-02
partial.cor = 0.313
p = 3.07e-09
partial.cor = 0.313
p = 3.38e-09
partial.cor = 0.066
p = 2.20e-01
partial.cor = 0.19
p = 4.34e-04
Infiltration Level
D
PRC1 Expression Level
cor = 0.199
p = 2e-04
partial.cor = 0.459
p = 2.31e-19
partial.cor = 0.285
p = 8.00e-08
partial.cor = 0.284
p = 8.23e-08
partial.cor = 0.397
p = 2.35e-14
partial.cor = 0.332
p = 2.60e-10
partial.cor = 0.401
p = 1.50e-14
Infiltration Level
E
RNASEH2A Expression Level
cor = 0.243
p = 4.84e-06
partial.cor = 0.304
p = 8.31e-09
partial.cor = 0.228
p = 2.02e-05
partial.cor = 0.041
p = 4.53e-01
partial.cor = 0.137
p = 1.13e-02
partial.cor = 0.05
p = 3.50e-01
partial.cor = 0.244
p = 5.34e-06
Infiltration Level
F
RRM2 Expression Level
cor = 0.133
p = 1.31e-02
partial.cor = 0.436
p = 2.22e-17
partial.cor = 0.302
p = 1.19e-08
partial.cor = 0.134
p = 1.29e-02
partial.cor = 0.279
p = 1.60e-07
partial.cor = 0.252
p = 2.18e-06
partial.cor = 0.385
p = 1.99e-13
Infiltration Level
G
SPATS2 Expression Level
cor = 0.081
p = 1.34e-01
partial.cor = 0.424
p = 1.90e-16
partial.cor = 0.256
p = 1.59e-06
partial.cor = 0.311
p = 4.00e-09
partial.cor = 0.43
p = 9.33e-17
partial.cor = 0.362
p = 3.93e-12
partial.cor = 0.383
p = 2.41e-13
Infiltration Level
H
TARBP1 Expression Level
cor = 0.142
p = 8.05e-03
partial.cor = 0.06
p = 2.63e-01
partial.cor = −0.146
p = 6.74e-03
partial.cor = 0.267
p = 5.25e-07
partial.cor = 0.109
p = 4.40e-02
partial.cor = −0.023
p = 6.66e-01
partial.cor = −0.027
p = 6.17e-01
Infiltration Level

Supplementary Figure 9. Correlation between levels of hub genes and immune cell infiltration. Correlation between *MCM3* (A), *MCM6* (B), *NT5DC2* (C), *PRC1* (D), *RNASEH2A* (E), *RRM2* (F), *SPATS2* (G), and *TARBP1* (H) levels and immune cell infiltration in hepatocellular carcinoma (HCC) tissues. Each dot represents a sample in the TCGA cohort.
