# Supplementary material for: Identification of hub genes in hepatocellular carcinoma using integrated bioinformatic analysis
Source: Aging (Albany NY). 2020 Mar 26;12(6):5439–68. doi: 10.18632/aging.102969 (PMC7138582; doi:10.18632/aging.102969)
Supplement: Supplementary Tables [file aging-12-102969-s003..pdf]

SUPPLEMENTARY TABLES

Please browse Full Text version to see the data of Supplementary Table 1.

Supplementary Table 1. List of 176 common upregulated genes among hepatocellular carcinoma datasets.

Supplementary Table 2. List of 57 common upregulated genes with higher protein levels among hepatocellular carcinoma datasets.

	Gene	
CLGN	MCAM	MCM4
GPNMB	PODXL	MAP2
AKR1B10	RFC4	MSH2
KPNA2	SPATS2	LRRC1
COL15A1	LAMC1	RNASEH2A
CDK1	TARBP1	COL4A1
FABP5	STMN1	CAP2
SORT1	TP53I3	GPC3
SULT1C2	COL4A2	MCM6
ENAH	IGF2BP2	MUC13
MAD2L1	SRXN1	SPARC
ATP6V1C1	PRC1	LAMA4
MCM3	LGALS3	PLVAP
NEDD4L	RRM2	PEG10
THY1	G6PD	HKDC1
CAPG	MDK	DTNA
ROBO1	FEN1	ACSL4
MPZL1	NT5DC2	TP53BP2
ITGA6	MCM2	PSPH

Please browse Full Text version to see the data of Supplementary Table 3.

Supplementary Table 3. Statistical analysis (*p*-values) of the expression of 12 upregulated genes comparing different cancer stages/tumor grades.

Supplementary Table 4. Primer sequences used in this study.

Type	Gene	Sequence
Primers	MCM3	F 5’ TCTGGGACCTTCAGGACTGT 3’
		R 5’ TTGATGTCCCCACGGATGTG 3’
	NT5DC2	F 5’ AGGGGATGAGACGTTTGCTG 3’
		R 5’ AAACAGGTTTCCCTGCCGAT 3’
	SPATS2	F 5’ CTTCGGGAGGTATTGCCAGG 3’
		R 5’ GCTCCATGCTGACTCCAGTT 3’
	RNASEH2A	F 5’ AGACAATACAGGCCGCTGTC 3’
		R 5’ GGGTCTTTGAGTCTGCCACT 3’
	TARBP1	F 5’ TGGGAAACAGGAGAAAACTGCT 3’
		R 5’ AGCAAGGGCATGTAGGCATT 3’

Supplementary Table 5. Antibodies used in this study.

Gene	Antibody
MCM3	1:50 for IHC 1:1000 for WB, from Proteintech, Chicago, IL, USA
NT5DC2	1:50 for IHC 1:1000 for WB, from Bioss, Beijing, China
SPATS2	1:50 for IHC 1:1000 for WB, from Bioss, Beijing, China
RNASEH2A	1:50 for IHC 1:1000 for WB, from Proteintech, Chicago, IL, USA
TARBP1	1:1000 for WB, from Abcam, Cambridge, UK
RRM2	1:50 for IHC, from Proteintech, Chicago, IL, USA
LRRC1	1:50 for IHC, from Proteintech, Chicago, IL, USA
