# Supplementary material for: Identification of hub genes in hepatocellular carcinoma using integrated bioinformatic analysis
Source: Aging (Albany NY). 2020 Mar 26;12(6):5439–68. doi: 10.18632/aging.102969 (PMC7138582; doi:10.18632/aging.102969)
Supplement: Supplementary Table 1 [file aging-12-102969-s001..docx]

**Supplementary Table 1. List of 176 common upregulated genes among hepatocellular carcinoma datasets.**

| **Gene** |
| --- |
| \| **CCNB1** \| \| --- \| \| **SMYD3** \| \| **FOXM1** \| \| **CDK1** \| \| **MAD2L1** \| \| **ATP6V1C1** \| \| **SOX9** \| \| **KIF4A** \| \| **SQLE** \| \| **TCF19** \| \| **ACLY** \| \| **TYMS** \| \| **TMEM106C** \| \| **MELK** \| \| **PTP4A3** \| \| **PODXL** \| \| **RBP7** \| \| **NDC80** \| \| **OIP5** \| \| **CCNA2** \| \| **STMN1** \| \| **CKS2** \| \| **TP53I3** \| \| **KIF23** \| \| **ANXA2** \| \| **SERPINI1** \| \| **CCNB2** \| \| **PRC1** \| \| **LGALS3** \| \| **CENPW** \| \| **G6PD** \| \| **TXNRD1** \| \| **MCM2** \| \| **MCM4** \| \| **LRRC1** \| \| **RAP2A** \| \| **GPC3** \| \| **CENPK** \| \| **DTNA** \| \| **LAPTM4B** \| \| **NEK2** \| \| **FANCD2** \| \| **PSPH** \| \| **B3GNT5** \| \| **C1orf106** \| \| **CLGN** \| \| **SOX4** \| \| **GPNMB** \| \| **AKR1B10** \| \| **EZH2** \| \| **ENAH** \| \| **CAPG** \| \| **ROBO1** \| \| **MPZL1** \| \| **MCAM** \| \| **SFN** \| \| **CDC20** \| \| **RFC4** \| \| **KDELR3** \| \| **BUB1** \| \| **TRIP13** \| \| **RGS5** \| \| **UBE2S** \| \| **UBE2C** \| \| **MND1** \| \| **ACSM1** \| \| **RRM2** \| \| **TOP2A** \| \| **FEN1** \| \| **HELLS** \| \| **FANCI** \| \| **SPARCL1** \| \| **MAP2** \| \| **S100A10** \| \| **CAP2** \| \| **FAM83D** \| \| **MCM6** \| \| **LCN2** \| \| **SPARC** \| \| **PEG10** \| \| **CTHRC1** \| \| **CDKN2A** \| \| **NOTCH3** \| \| **TRNP1** \| \| **CDKN3** \| \| **CENPM** \| \| **CENPF** \| \| **KRT23** \| \| **CDCA8** \| \| **TKT** \| \| **TPX2** \| \| **C15orf48** \| \| **NSMCE2** \| \| **GINS1** \| \| **ZIC2** \| \| **KPNA2** \| \| **ANLN** \| \| **BIRC5** \| \| **FABP5** \| \| **SORT1** \| \| **RACGAP1** \| \| **CDC6** \| \| **SULT1C2** \| \| **AURKA** \| \| **AURKB** \| \| **PAFAH1B3** \| \| **C1orf198** \| \| **NEDD4L** \| \| **BEX2** \| \| **EPS8L3** \| \| **THY1** \| \| **SPP1** \| \| **ITGA6** \| \| **ZWINT** \| \| **NUF2** \| \| **PTTG1** \| \| **TARBP1** \| \| **CDCA5** \| \| **UBE2T** \| \| **ECT2** \| \| **IQGAP3** \| \| **COL4A2** \| \| **IGF2BP2** \| \| **PDGFA** \| \| **NT5DC2** \| \| **DLGAP5** \| \| **RAD51AP1** \| \| **COL4A1** \| \| **MKI67** \| \| **LYZ** \| \| **CDKN2C** \| \| **MUC13** \| \| **PLVAP** \| \| **KIAA0101** \| \| **ACSL4** \| \| **TP53BP2** \| \| **SPINK1** \| \| **RFX5** \| \| **FLVCR1** \| \| **COL15A1** \| \| **TRIM31** \| \| **CDC25C** \| \| **TUBA1B** \| \| **MCM3** \| \| **NCAPG2** \| \| **KIF2C** \| \| **VWF** \| \| **PBK** \| \| **SPATS2** \| \| **LAMC1** \| \| **NCAPH** \| \| **TK1** \| \| **ASPM** \| \| **CDCA3** \| \| **SRXN1** \| \| **LOXL2** \| \| **ATAD2** \| \| **PEA15** \| \| **STXBP6** \| \| **CKAP2** \| \| **MDK** \| \| **SPC25** \| \| **MSH2** \| \| **BUB1B** \| \| **OLFML2B** \| \| **HJURP** \| \| **RNASEH2A** \| \| **DTL** \| \| **HMMR** \| \| **GMNN** \| \| **KIF20A** \| \| **LAMA4** \| \| **RNF157** \| \| **HKDC1** \| \| **NCAPG** \| \| **NUSAP1** \| |
