# Supplementary material for: Identification of hub genes in hepatocellular carcinoma using integrated bioinformatic analysis
Source: Aging (Albany NY). 2020 Mar 26;12(6):5439–68. doi: 10.18632/aging.102969 (PMC7138582; doi:10.18632/aging.102969)
Supplement: Supplementary Table 3 [file aging-12-102969-s002..docx]

**Supplementary Table 3****. Statistical analysis (*p*-values) of the expression of 12 upregulated genes comparing different cancer stages/tumor grades.**

| **Gene** | **stages/grade** | **Comparison** | **Statistical significance** |
| --- | --- | --- | --- |
| **CDK1** | **cancer stages** | **Normal-vs-Stage1** | **<1E-12** |
|  |  | **Normal-vs-Stage2** | **1.96799998519737E-09** |
|  |  | **Normal-vs-Stage3** | **2.0140555889725E-12** |
|  |  | **Normal-vs-Stage4** | **4.641900E-02** |
|  |  | **Stage1-vs-Stage2** | **5.836200E-03** |
|  |  | **Stage1-vs-Stage3** | **1.68258999999615E-05** |
|  |  | **Stage1-vs-Stage4** | **7.378200E-01** |
|  |  | **Stage2-vs-Stage3** | **2.101400E-01** |
|  |  | **Stage2-vs-Stage4** | **4.135500E-02** |
|  |  | **Stage3-vs-Stage4** | **4.638800E-03** |
|  | **tumor grade** | **Normal-vs-Grade 1** | **1.418260E-04** |
|  |  | **Normal-vs-Grade 2** | **1.62636570877339E-12** |
|  |  | **Normal-vs-Grade 3** | **1.62447832963153E-12** |
|  |  | **Normal-vs-Grade 4** | **7.203500E-04** |
|  |  | **Grade 1-vs-Grade 2** | **3.644600E-01** |
|  |  | **Grade 1-vs-Grade 3** | **2.297100E-03** |
|  |  | **Grade 1-vs-Grade 4** | **1.570350E-01** |
|  |  | **Grade 2-vs-Grade 3** | **3.142900E-03** |
|  |  | **Grade 2-vs-Grade 4** | **3.621000E-01** |
|  |  | **Grade 3-vs-Grade 4** | **7.710200E-01** |
| **FEN1** | **cancer stages** | **Normal-vs-Stage1** | **<1E-12** |
|  |  | **Normal-vs-Stage2** | **8.43769498715119E-14** |
|  |  | **Normal-vs-Stage3** | **<1E-12** |
|  |  | **Normal-vs-Stage4** | **5.258200E-02** |
|  |  | **Stage1-vs-Stage2** | **1.499700E-02** |
|  |  | **Stage1-vs-Stage3** | **6.162600E-04** |
|  |  | **Stage1-vs-Stage4** | **9.834000E-01** |
|  |  | **Stage2-vs-Stage3** | **5.513800E-01** |
|  |  | **Stage2-vs-Stage4** | **4.622400E-01** |
|  |  | **Stage3-vs-Stage4** | **2.815600E-01** |
|  | **tumor grade** | **Normal-vs-Grade 1** | **6.7767999989421E-08** |
|  |  | **Normal-vs-Grade 2** | **1.62436730732907E-12** |
|  |  |  |  |
|  |  | **Normal-vs-Grade 3** | **<1E-12** |
|  |  | **Normal-vs-Grade 4** | **4.443799999998E-05** |
|  |  | Grade 1-vs-Grade 2 | 5.184000E-01 |
|  |  | **Grade 1-vs-Grade 3** | **1.298730E-03** |
|  |  | **Grade 1-vs-Grade 4** | **4.347700E-02** |
|  |  | **Grade 2-vs-Grade 3** | **5.182500E-04** |
|  |  | Grade 2-vs-Grade 4 | 6.641700E-02 |
|  |  | Grade 3-vs-Grade 4 | 9.092400E-01 |
| **KPNA2** | **cancer stages** | **Normal-vs-Stage1** | **1.62447832963153E-12** |
|  |  | **Normal-vs-Stage2** | **2.30915286891786E-12** |
|  |  | **Normal-vs-Stage3** | **6.66133814775094E-15** |
|  |  | **Normal-vs-Stage4** | **4.844400E-02** |
|  |  | **Stage1-vs-Stage2** | **2.656900E-03** |
|  |  | **Stage1-vs-Stage3** | **3.59380000000131E-05** |
|  |  | Stage1-vs-Stage4 | 9.556800E-01 |
|  |  | Stage2-vs-Stage3 | 3.223800E-01 |
|  |  | Stage2-vs-Stage4 | 3.680000E-01 |
|  |  | Stage3-vs-Stage4 | 2.045400E-01 |
|  | **tumor grade** | **Normal-vs-Grade 1** | **8.32570000053323E-07** |
|  |  | **Normal-vs-Grade 2** | **<1E-12** |
|  |  | **Normal-vs-Grade 3** | **1.62447832963153E-12** |
|  |  | **Normal-vs-Grade 4** | **1.226560E-04** |
|  |  | Grade 1-vs-Grade 2 | 3.008400E-01 |
|  |  | **Grade 1-vs-Grade 3** | **1.078660E-03** |
|  |  | Grade 1-vs-Grade 4 | 7.450400E-02 |
|  |  | **Grade 2-vs-Grade 3** | **2.355000E-03** |
|  |  | Grade 2-vs-Grade 4 | 1.852370E-01 |
|  |  | Grade 3-vs-Grade 4 | 7.871000E-01 |
| **LRRC1** | **cancer stages** | **Normal-vs-Stage1** | **<1E-12** |
|  |  | **Normal-vs-Stage2** | **1.33330013696309E-11** |
|  |  | **Normal-vs-Stage3** | **3.91989773973478E-11** |
|  |  | **Normal-vs-Stage4** | **4.164300E-02** |
|  |  | Stage1-vs-Stage2 | 1.803400E-01 |
|  |  | **Stage1-vs-Stage3** | **8.837800E-03** |
|  |  | Stage1-vs-Stage4 | 7.731600E-01 |
|  |  | Stage2-vs-Stage3 | 1.468160E-01 |
|  |  | Stage2-vs-Stage4 | 5.084000E-01 |
|  |  | Stage3-vs-Stage4 | 5.768000E-02 |
|  | **tumor grade** |  |  |
|  |  | **Normal-vs-Grade 1** | **1.043830E-03** |
|  |  | **Normal-vs-Grade 2** | **1.62592161956354E-12** |
|  |  | **Normal-vs-Grade 3** | **<1E-12** |
|  |  | **Normal-vs-Grade 4** | **1.159600E-03** |
|  |  | Grade 1-vs-Grade 2 | 1.105190E-01 |
|  |  | **Grade 1-vs-Grade 3** | **3.364600E-04** |
|  |  | **Grade 1-vs-Grade 4** | **2.237000E-03** |
|  |  | **Grade 2-vs-Grade 3** | **7.777800E-03** |
|  |  | **Grade 2-vs-Grade 4** | **1.885970E-02** |
|  |  | Grade 3-vs-Grade 4 | 1.658760E-01 |
| **MCM3** | **cancer stages** | **Normal-vs-Stage1** | **<1E-12** |
|  |  | **Normal-vs-Stage2** | **1.77569070558548E-12** |
|  |  | **Normal-vs-Stage3** | **1.62447832963153E-12** |
|  |  | Normal-vs-Stage4 | 5.841900E-02 |
|  |  | **Stage1-vs-Stage2** | **1.121770E-02** |
|  |  | **Stage1-vs-Stage3** | **8.04890000000169E-05** |
|  |  | Stage1-vs-Stage4 | 5.739600E-01 |
|  |  | Stage2-vs-Stage3 | 4.717400E-01 |
|  |  | Stage2-vs-Stage4 | 2.419400E-01 |
|  |  | Stage3-vs-Stage4 | 1.031380E-01 |
|  | **tumor grade** | **Normal-vs-Grade 1** | **1.80894999999293E-05** |
|  |  | **Normal-vs-Grade 2** | **1.62447832963153E-12** |
|  |  | **Normal-vs-Grade 3** | **<1E-12** |
|  |  | **Normal-vs-Grade 4** | **3.337100E-03** |
|  |  | Grade 1-vs-Grade 2 | 8.624400E-01 |
|  |  | **Grade 1-vs-Grade 3** | **9.136900E-03** |
|  |  | Grade 1-vs-Grade 4 | 1.571360E-01 |
|  |  | **Grade 2-vs-Grade 3** | **1.758360E-04** |
|  |  | Grade 2-vs-Grade 4 | 1.784440E-01 |
|  |  | Grade 3-vs-Grade 4 | 8.429400E-01 |
| **MCM6** | **cancer stages** | **Normal-vs-Stage1** | **<1E-12** |
|  |  | **Normal-vs-Stage2** | **2.80030443278179E-11** |
|  |  | **Normal-vs-Stage3** | **1.11022302462516E-16** |
|  |  | Normal-vs-Stage4 | 1.243980E-01 |
|  |  | **Stage1-vs-Stage2** | **4.760100E-03** |
|  |  | **Stage1-vs-Stage3** | **2.49099999999336E-05** |
|  |  | Stage1-vs-Stage4 | 8.087600E-01 |
|  |  | Stage2-vs-Stage3 | 5.106200E-01 |
|  |  | Stage2-vs-Stage4 | 3.530000E-01 |
|  |  | Stage3-vs-Stage4 | 1.611400E-01 |
|  | **tumor grade** | **Normal-vs-Grade 1** | **1.68170000003354E-06** |
|  |  | **Normal-vs-Grade 2** | **1.62436730732907E-12** |
|  |  | **Normal-vs-Grade 3** | **<1E-12** |
|  |  | **Normal-vs-Grade 4** | **1.356580E-03** |
|  |  | Grade 1-vs-Grade 2 | 8.430200E-01 |
|  |  | **Grade 1-vs-Grade 3** | **2.657000E-03** |
|  |  | Grade 1-vs-Grade 4 | 2.013200E-01 |
|  |  | **Grade 2-vs-Grade 3** | **1.198190E-04** |
|  |  | Grade 2-vs-Grade 4 | 1.233560E-01 |
|  |  | Grade 3-vs-Grade 4 | 6.997400E-01 |
| **NT5DC2** | **cancer stages** | **Normal-vs-Stage1** | **1.62503344114384E-12** |
|  |  | **Normal-vs-Stage2** | **2.20100000003498E-07** |
|  |  | **Normal-vs-Stage3** | **5.85470000036281E-07** |
|  |  | Normal-vs-Stage4 | 8.124100E-02 |
|  |  | **Stage1-vs-Stage2** | **8.758400E-03** |
|  |  | **Stage1-vs-Stage3** | **1.122280E-03** |
|  |  | Stage1-vs-Stage4 | 9.534400E-01 |
|  |  | Stage2-vs-Stage3 | 2.006000E-01 |
|  |  | Stage2-vs-Stage4 | 1.221030E-01 |
|  |  | **Stage3-vs-Stage4** | **1.740000E-02** |
|  | **tumor grade** | **Normal-vs-Grade 1** | **5.83880000015746E-07** |
|  |  | **Normal-vs-Grade 2** | **8.7859719499761E-12** |
|  |  | **Normal-vs-Grade 3** | **7.22439996714286E-09** |
|  |  | **Normal-vs-Grade 4** | **3.061800E-02** |
|  |  | **Grade 1-vs-Grade 2** | **3.539900E-03** |
|  |  | **Grade 1-vs-Grade 3** | **1.198990E-04** |
|  |  | Grade 1-vs-Grade 4 | 1.190610E-01 |
|  |  | **Grade 2-vs-Grade 3** | **3.794400E-02** |
|  |  | Grade 2-vs-Grade 4 | 2.487800E-01 |
|  |  | Grade 3-vs-Grade 4 | 9.916800E-01 |
| **PRC1** | **cancer stages** | **Normal-vs-Stage1** | **1.62447832963153E-12** |
|  |  | **Normal-vs-Stage2** | **5.76427794385381E-12** |
|  |  | **Normal-vs-Stage3** | **1.68576264059084E-12** |
|  |  | Normal-vs-Stage4 | 1.290590E-01 |
|  |  | **Stage1-vs-Stage2** | **1.920250E-02** |
|  |  | **Stage1-vs-Stage3** | **6.23499999999888E-05** |
|  |  | Stage1-vs-Stage4 | 6.588200E-01 |
|  |  | Stage2-vs-Stage3 | 8.096300E-02 |
|  |  | Stage2-vs-Stage4 | 8.498800E-01 |
|  |  | Stage3-vs-Stage4 | 4.490200E-01 |
|  | **tumor grade** | **Normal-vs-Grade 1** | **1.177260E-04** |
|  |  | **Normal-vs-Grade 2** | **1.62447832963153E-12** |
|  |  | **Normal-vs-Grade 3** | **1.62447832963153E-12** |
|  |  | **Normal-vs-Grade 4** | **2.079500E-03** |
|  |  | Grade 1-vs-Grade 2 | 6.412800E-01 |
|  |  | **Grade 1-vs-Grade 3** | **3.764700E-03** |
|  |  | Grade 1-vs-Grade 4 | 2.412000E-01 |
|  |  | **Grade 2-vs-Grade 3** | **1.223050E-04** |
|  |  | Grade 2-vs-Grade 4 | 1.558990E-01 |
|  |  | Grade 3-vs-Grade 4 | 6.561000E-01 |
| **RNASEH2A** | **cancer stages** | **Normal-vs-Stage1** | **<1E-12** |
|  |  | **Normal-vs-Stage2** | **2.22044604925031E-16** |
|  |  | **Normal-vs-Stage3** | **1.62458935193399E-12** |
|  |  | **Normal-vs-Stage4** | **9.001900E-03** |
|  |  | **Stage1-vs-Stage2** | **1.639800E-02** |
|  |  | **Stage1-vs-Stage3** | **3.581000E-04** |
|  |  | Stage1-vs-Stage4 | 7.244200E-01 |
|  |  | Stage2-vs-Stage3 | 1.960430E-01 |
|  |  | Stage2-vs-Stage4 | 3.449600E-01 |
|  |  | **Stage3-vs-Stage4** | **1.470010E-02** |
|  | **tumor grade** | **Normal-vs-Grade 1** | **7.09290000067142E-08** |
|  |  | **Normal-vs-Grade 2** | **1.62447832963153E-12** |
|  |  | **Normal-vs-Grade 3** | **1.62447832963153E-12** |
|  |  | **Normal-vs-Grade 4** | **1.437770E-04** |
|  |  | Grade 1-vs-Grade 2 | 6.574000E-01 |
|  |  | **Grade1-vs-Grade 3** | **5.683200E-03** |
|  |  | Grade 1-vs-Grade 4 | 1.485440E-01 |
|  |  | **Grade2-vs-Grade 3** | **3.754500E-04** |
|  |  | Grade 2-vs-Grade 4 | 1.038960E-01 |
|  |  | Grade 3-vs-Grade 4 | 8.931000E-01 |
| **RRM2** | **cancer stages** | **Normal-vs-Stage1** | **1.62436730732907E-12** |
|  |  | **Normal-vs-Stage2** | **1.62458935193399E-12** |
|  |  | **Normal-vs-Stage3** | **3.01260016932758E-10** |
|  |  | Normal-vs-Stage4 | 1.173550E-01 |
|  |  | **Stage1-vs-Stage2** | **2.324300E-03** |
|  |  | **Stage1-vs-Stage3** | **1.883380E-03** |
|  |  | Stage1-vs-Stage4 | 7.355200E-01 |
|  |  | Stage2-vs-Stage3 | 2.270200E-01 |
|  |  | Stage2-vs-Stage4 | 5.157000E-01 |
|  |  | Stage3-vs-Stage4 | 4.589600E-01 |
|  | **tumor grade** | **Normal-vs-Grade 1** | **1.543430E-03** |
|  |  | **Normal-vs-Grade 2** | **1.62447832963153E-12** |
|  |  | **Normal-vs-Grade 3** | **1.62447832963153E-12** |
|  |  | **Normal-vs-Grade 4** | **1.65399999996207E-06** |
|  |  | Grade 1-vs-Grade 2 | 8.268800E-01 |
|  |  | Grade 1-vs-Grade 3 | 1.687410E-01 |
|  |  | Grade 1-vs-Grade 4 | 2.133400E-01 |
|  |  | **Grade2-vs-Grade 3** | **2.776500E-04** |
|  |  | Grade 2-vs-Grade 4 | 7.547700E-02 |
|  |  | Grade 3-vs-Grade 4 | 9.318200E-01 |
| **SPATS2** | **cancer stages** | **Normal-vs-Stage1** | **<1E-12** |
|  |  | **Normal-vs-Stage2** | **1.65334412827178E-12** |
|  |  | **Normal-vs-Stage3** | **1.62436730732907E-12** |
|  |  | Normal-vs-Stage4 | 9.530400E-02 |
|  |  | **Stage1-vs-Stage2** | **8.298900E-03** |
|  |  | **Stage1-vs-Stage3** | **1.179610E-04** |
|  |  | Stage1-vs-Stage4 | 6.705800E-01 |
|  |  | Stage2-vs-Stage3 | 3.565600E-01 |
|  |  | Stage2-vs-Stage4 | 6.241200E-01 |
|  |  | Stage3-vs-Stage4 | 3.888400E-01 |
|  | **tumor grade** | **Normal-vs-Grade 1** | **9.51940000049056E-07** |
|  |  | **Normal-vs-Grade 2** | **<1E-12** |
|  |  | **Normal-vs-Grade 3** | **<1E-12** |
|  |  | **Normal-vs-Grade 4** | **4.879700E-03** |
|  |  | Grade 1-vs-Grade 2 | 2.331600E-01 |
|  |  | **Grade1-vs-Grade 3** | **4.762700E-04** |
|  |  | Grade 1-vs-Grade 4 | 1.013240E-01 |
|  |  | **Grade2-vs-Grade 3** | **1.594540E-04** |
|  |  | Grade 2-vs-Grade 4 | 1.741560E-01 |
|  |  | Grade 3-vs-Grade 4 | 6.109400E-01 |
| **TARBP1** | **cancer stages** | **Normal-vs-Stage1** | **<1E-12** |
|  |  | **Normal-vs-Stage2** | **1.62458935193399E-12** |
|  |  | **Normal-vs-Stage3** | **1.62458935193399E-12** |
|  |  | **Normal-vs-Stage4** | **2.948200E-02** |
|  |  | Stage1-vs-Stage2 | 2.391400E-01 |
|  |  | **Stage1-vs-Stage3** | **4.114100E-02** |
|  |  | Stage1-vs-Stage4 | 4.604400E-01 |
|  |  | Stage2-vs-Stage3 | 4.187000E-01 |
|  |  | Stage2-vs-Stage4 | 7.989800E-01 |
|  |  | Stage3-vs-Stage4 | 9.589800E-01 |
|  | **tumor grade** | **Normal-vs-Grade 1** | **2.53969956176547E-10** |
|  |  | **Normal-vs-Grade 2** | **<1E-12** |
|  |  | **Normal-vs-Grade 3** | **1.62447832963153E-12** |
|  |  | **Normal-vs-Grade 4** | **3.622400E-03** |
|  |  | Grade 1-vs-Grade 2 | 5.333200E-01 |
|  |  | **Grade1-vs-Grade 3** | **9.864200E-03** |
|  |  | Grade 1-vs-Grade 4 | 2.186400E-01 |
|  |  | **Grade2-vs-Grade 3** | **4.495100E-03** |
|  |  | Grade 2-vs-Grade 4 | 3.169800E-01 |
|  |  | Grade 3-vs-Grade 4 | 8.357000E-01 |
